# Therapeutic Effects of Glycyrrhizic Acid on Dry Eye Disease: Targeting Pyroptosis, Oxidative Stress, and Epithelial Barrier Dysfunction

**DOI:** 10.3390/ijms27094153

**Published:** 2026-05-06

**Authors:** Yiran Chu, Chengxiao Zhang, Zeying Chen, Qi Zhang, Yun Tang, Jiaxuan Jiang, Kai Hu

**Affiliations:** 1 Department of Ophthalmology, Nanjing Drum Tower Hospital, Affiliated Hospital of Medical School, Nanjing University, 321 Zhongshan Road, Nanjing 210008, China; 522023350165@smail.nju.edu.cn (Y.C.); cxzhangfernanda@foxmail.com (C.Z.); 522023350202@smail.nju.edu.cn (Z.C.); 622023350069@smail.nju.edu.cn (Y.T.); 2 Department of Ophthalmology, Nanjing Drum Tower Hospital Clinical College, Nanjing University of Chinese Medicine, 321 Zhongshan Road, Nanjing 210008, China; 20231883@njucm.edu.cn

**Keywords:** dry eye disease, glycyrrhizic acid, pyroptosis, oxidative stress, epithelial barrier dysfunction

## Abstract

Dry eye disease (DED) is a common ocular surface disorder characterized by instability of the tear film, inflammatory responses, and epithelial damage, and therapeutic interventions directed at these fundamental pathogenetic processes are still insufficient. This research aimed to evaluate the medicinal efficacy of glycyrrhizic acid (GA) and to unravel the underlying molecular pathways through which it exerts its protective role in DED. A benzalkonium chloride-induced mouse model and a hyperosmolarity-induced human corneal epithelial cell model were established. Corneal epithelial injury, tear secretion, and goblet cell density were evaluated in vivo, while cellular responses and related signaling pathways were examined using RT-qPCR, Western blotting, flow cytometry, and immunofluorescence. GA treatment alleviated corneal epithelial damage, increased tear secretion, and improved goblet cell density in mice. In vitro, GA reduced inflammatory responses, as evidenced by decreased tumor necrosis factor-α (TNF-α) expression, and helped preserve epithelial barrier integrity, accompanied by reduced matrix metalloprotease 9 (MMP9) levels. Further analysis suggested that GA suppressed pyroptosis through regulation of the high mobility group box 1 (HMGB1)/lysosomal membrane permeabilization (LMP)/cathepsin B (CTSB) pathway and attenuated oxidative stress via activation of the nuclear factor erythroid 2–related factor 2 (Nrf2)/heme oxygenase-1 (HO-1)/NAD (P)H:quinone oxidoreductase 1 (NQO1) axis. In addition, GA improved mitochondrial function, as indicated by decreased reactive oxygen species levels, restored membrane potential, and enhanced adenosine triphosphate (ATP) production. Taken together, these findings indicate that GA may alleviate hyperosmolarity-induced DED by modulating inflammation, oxidative stress, mitochondrial dysfunction, and epithelial barrier damage, underscoring its viability as a remedial candidate.

## 1. Introduction

Dry eye disease (DED) is a prevalent ocular surface disorder characterized by disruption of tear film equilibrium, inflammatory processes, and epithelial damage, which can lead to symptoms such as dryness, irritation, and visual disturbance [1,2,3]. The global prevalence of DED ranges from 5% to 50% [4,5,6,7,8], with a substantial impact on patients’ quality of life [9,10,11], including visual disturbance, discomfort, and reduced daily functioning [12,13,14], as well as associated psychological distress such as anxiety and depression [15,16].

Increasing evidence indicates that DED is driven by a complex interplay of inflammatory responses, oxidative stress, and dysregulated cell death [17,18,19]. However, the overall efficacy of the existing treatment methods is limited, and it is difficult to comprehensively intervene in the multi-factor pathological process. Artificial tears, topical immunomodulators (such as cyclosporine), and corticosteroids are commonly used clinical treatments. While artificial tears provide merely transient symptomatic comfort, the long-term administration of cyclosporine and corticosteroids is frequently associated with the development of undesirable secondary effects [20,21,22]. In addition, drugs approved by the FDA in recent years also include lymphocyte function-associated antigen 1 (LFA-1) antagonist lifitegrast eye drops and cholinergic receptor agonist varenicline nasal spray [23,24]. While these agents modulate immune responses or enhance lacrimation, there are still problems of adverse reactions such as ocular irritation and dysgeusia and individual differences in efficacy [4,25]. Given the limitations of existing therapies, it is imperative to explore multi-target therapeutic paradigms that surpass the limitations of current treatments.

The high mobility group box 1 (HMGB1), a stress-responsive nuclear-binding protein, can induce lysosomal membrane permeabilization (LMP) [26], leading to the release of lysosomal enzymes such as cathepsin B (CTSB) [27,28] and triggering pyroptosis, a pro-inflammatory form of programmed cell death. This pathway has been reported to be activated in DED and leads to ocular surface damage [29]. However, effective strategies to modulate HMGB1/LMP/CTSB-mediated pyroptosis in DED remain limited.

Oxidative stress is another central feature of DED pathogenesis [30]. It is characterized by the imbalance between the production of intracellular reactive oxygen species (ROS) and the capacity of the endogenous antioxidant defense system to neutralize them [31]. In the dry eye state, hyperosmolarity-induced mitochondrial dysfunction drives the aberrant production of ROS. Oxidative stress arises on the ocular surface when the production of ROS surpasses the scavenging capacity of endogenous antioxidant enzymes, such as superoxide dismutase (SOD) and glutathione peroxidase (GPX) [32].

Oxidative insults serve to compromise the meibomian gland, lacrimal gland, and ocular surface epithelia via the pathway of lipid peroxidation and other mechanisms, destroying their structural integrity [33,34]. Furthermore, oxidative stress acts as a pivotal upstream signal that mobilizes the NF-κB pathway, promoting the release of pro-inflammatory agents such as interleukin-1β (IL-1β) and tumor necrosis factor-α (TNF-α) and escalating localized damage into a systemic immune response [30,35]. The inflammatory response is enhanced through leukocyte recruitment and metabolism, which in turn further promotes the production of ROS [36]. This bidirectional feedback establishes a self-reinforcing vicious cycle [37], which transforms the pathological state of DED from an initial transient injury to a chronic, self-sustaining progressive process.

At the same time, impairment of endogenous antioxidant systems further exacerbates this imbalance [33]. Nuclear factor erythroid 2-related factor 2 (Nrf2) is a primary regulator of protective antioxidants, modulating the expression of proteins like heme oxygenase-1 (HO-1) and NAD (P)H:quinone oxidoreductase 1 (NQO1) [38,39]. The ability of Nrf2 induction to alleviate oxidative stress highlights its potential as a therapeutic target for ocular surface disorders [40,41,42]. Activating the Nrf2 pathway to restore redox balance and disrupt the vicious cycle driven by oxidative stress presents a promising treatment strategy.

Besides inflammation and oxidative stress, a compromised corneal epithelial barrier serves as a central driver in the breakdown of ocular surface homeostasis during DED progression [43,44]. Tight junction proteins, including zonula occludens-1 (ZO-1), are essential for maintaining epithelial integrity. Hyperosmotic stress, a hallmark of DED, has been shown to impair tight junctions through inflammatory mediators such as TNF-α and matrix metalloproteinase 9 (MMP9) [45,46], leading to barrier dysfunction. Therefore, preserving epithelial barrier function represents an important aspect of DED treatment.

Glycyrrhizic acid (GA), a bioactive compound derived from Glycyrrhiza, has been widely reported to exhibit anti-inflammatory, antioxidant, and cytoprotective effects [47,48]. In recent years, GA has shown effects in inhibiting HMGB1 activity and modulating multiple cellular processes, including oxidative stress and mitochondrial function. A study by Carol et al. demonstrated that treating adult patients with moderate DED with 2.5% glycyrrhizin eye drops twice daily for 28 days resulted in a significant improvement in tear break-up time (TBUT) and Schirmer scores, alongside a significant reduction in patient-reported symptoms [49]. However, the underlying mechanism of GA’s therapeutic effects in DED remains unreported.

Utilizing both in vitro and in vivo experimental frameworks, this study systematically interrogated the pharmacological efficacy and underlying molecular mechanisms of GA in treating DED. We found that GA mitigated ocular surface damage and increased aqueous tear secretion, while suppressing HMGB1/LMP/CTSB-mediated pyroptosis, reducing oxidative stress through activating the Nrf2 pathway, and preserving mitochondrial function and epithelial barrier integrity. These findings provide new insights into the multi-target effects of GA and underscore the remedial prospects of GA in addressing the pathological challenges of DED.

## 2. Results

### 2.1. Therapeutic Efficacy of GA on Ocular Surface Damage of DED Mice

**Figure 1:**
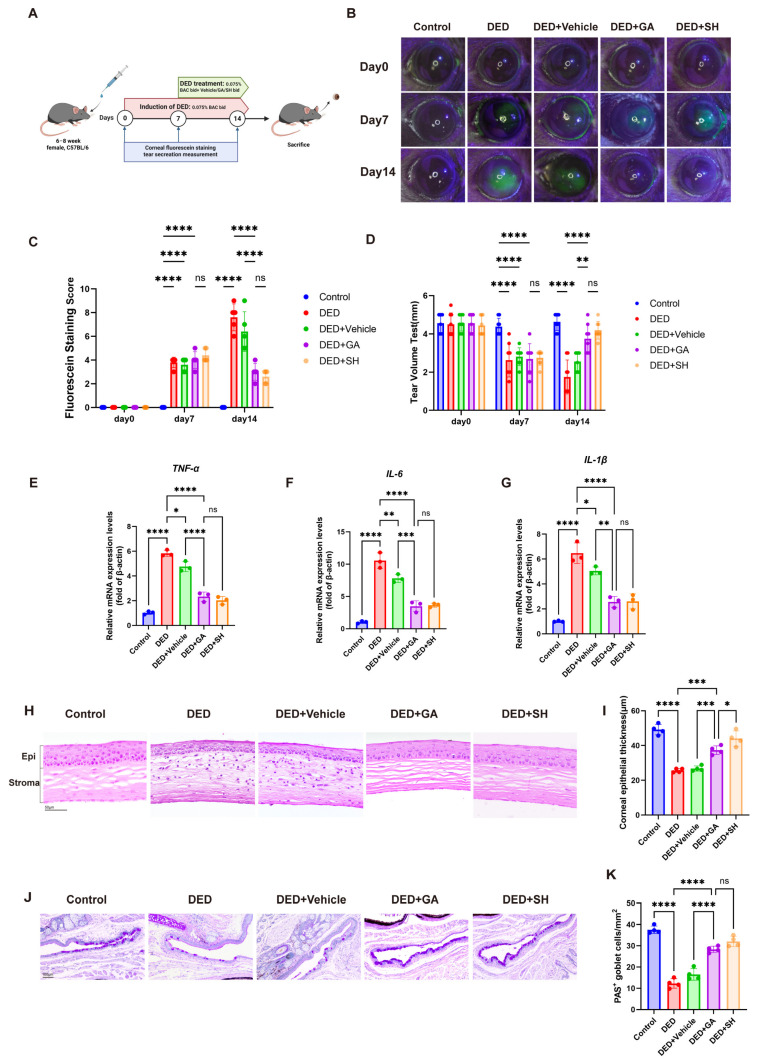
The therapeutic efficacy of GA eye drops in DED mice. (**A**) The diagram of DED modeling, treatment, and ocular assessment timeline. (**B**) Photographs of corneal fluorescein staining (CFS) of mice across all designated groups at days 0, 7, and 14. (**C**) Quantitative analysis of CFS scores (n = 5). (**D**) Measurement of tear volume (n = 5). (**E**–**G**) RT-qPCR was employed to determine the mRNA levels of key pro-inflammatory cytokines including *TNF-α*, *IL-1β*, and *IL-6*, within the corneal tissues (n = 3). (**H**) H&E staining of corneas in different groups (n = 4, Scale bar = 50 μm). (**I**) Measurement of corneal epithelial layer thickness (n = 4). (**J**,**K**) Morphological evaluation of the conjunctiva via PAS staining in each group of mice (Scale bar = 100 μm) and quantification of conjunctival goblet cell density (n = 4). The data were presented as mean ± SD of at least three independent experiments (ns, *p* > 0.05, * *p* < 0.05, ** *p* < 0.01, *** *p* < 0.001, **** *p* < 0.0001).

To evaluate the efficacy of GA eye drops in alleviating DED signs in mice, we established a BAC (0.075% benzalkonium chloride)-induced DED model. Mice in the DED group received BAC eye drops for 14 days. After a 7-day BAC induction period, mice in the DED + Vehicle (0.1% DMSO), DED + GA, and DED + SH (sodium hyaluronate) groups were treated with 0.1% DMSO, 4 mM GA, or SH eye drops, respectively, twice daily for 7 consecutive days (Figure 1A). SH served as a positive control to provide a clinically relevant benchmark for GA efficacy.

GA treatment restored corneal epithelial integrity, reduced CFS scores, and promoted tear secretion (Figure 1B–D), with effects comparable to SH. Since DED progression is associated with ocular surface inflammation, we evaluated *TNF-α*, *IL-1β*, and *IL-6* expression by RT-qPCR. GA inhibited transcription of these pro-inflammatory cytokines in corneal tissue (Figure 1E–G). HE staining of corneal sections revealed epithelial thinning and increased inflammatory cell infiltration in DED mice, while GA restored epithelial thickness and reduced inflammation in the corneal stroma (Figure 1H,I). Periodic Acid Schiff (PAS) staining and subsequent analysis showed that GA also rescued conjunctival goblet cell numbers (Figure 1J,K). These results demonstrate the potent capacity of GA to mitigate DED signs, thereby confirming its therapeutic potential.

### 2.2. GA Attenuates Hyperosmolarity-Induced Injury in HCECs

**Figure 2:**
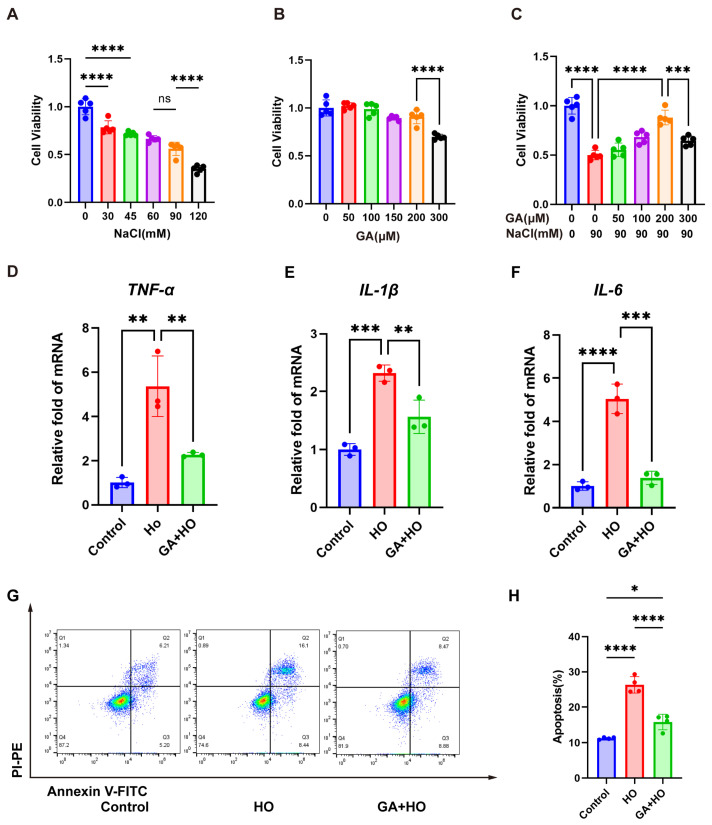
The protective efficacy of GA against hyperosmotic stress in HCECs. (**A**) Viability profiling of HCECs subjected to varied NaCl concentrations for 24 h (CCK-8 assay; n = 5). (**B**) Influences of a 24 h GA treatment at multiple concentrations on HCEC viability (CCK-8 assay; n = 5). (**C**) Protective effect of GA pretreatment at indicated concentrations (0–300 μM) against 90 mM NaCl-induced cytotoxicity in HCECs, determined via CCK-8 analysis (n = 5). (**D**–**F**) RT-qPCR was employed to determine the mRNA levels of key pro-inflammatory cytokines, including *TNF-α*, *IL-1β*, and *IL-6*, within the cells (n = 3). (**G**,**H**) Annexin V-FITC/PI-based flow cytometry was performed to measure HCEC death rates (n = 4) and quantification. The data were presented as mean ± SD of at least three independent experiments (ns, *p* > 0.05, * *p* < 0.05, ** *p* < 0.01, *** *p* < 0.001, **** *p* < 0.0001).

To induce hyperosmotic stress, human corneal epithelial cells (HCECs) were exposed to culture medium supplemented with a gradient of NaCl concentrations (0, 30, 45, 60, 90, and 120 mM) for a 24 h duration. Cell viability plummeted to roughly 50% at 90 mM NaCl and fell below 50% at 120 mM (Figure 2A). Consequently, 90 mM NaCl was selected to simulate hyperosmotic stress conditions characteristic of DED. HCECs were treated with GA at concentrations of 50, 100, 150, 200, and 300 μM for 24 h. As shown in Figure 2B, concentrations of 50, 100, 150, and 200 μM did not induce significant cytotoxicity. However, at a concentration of 300 μM, HCEC viability showed a notable decrease. Consequently, 200 μM was adopted as the highest concentration for GA intervention in the following in vitro experiments. To assess the protective effect of GA, HCECs were pretreated with GA at 50, 100, 200, and 300 μM for 24 h, followed by a 24 h exposure to hypertonic medium. The data indicated that while hyperosmotic treatment significantly reduced cell viability, GA could rescue it in a dose-dependent manner (Figure 2C). Furthermore, we observed that GA suppressed the mRNA expression of pro-inflammatory factors *TNF-α*, *IL-1β*, and *IL-6* induced by hyperosmolarity (Figure 2D–F) and reduced apoptosis, as verified through Annexin V-FITC/PI staining (Figure 2G,H).

### 2.3. GA Ameliorates Pyroptosis In Vitro and In Vivo by Downregulating the HMGB1/LMP/CTSB Pathway

Existing studies have implicated HMGB1, lysosomal membrane permeabilization (LMP), and pyroptosis in DED. Under hyperosmotic stress, HMGB1 translocates from the nucleus to the cytoplasm, triggering LMP. Increased LMP can activate Cathepsin B (CTSB), ultimately initiating cellular pyroptosis and further exacerbating ocular surface damage [29].

**Figure 3:**
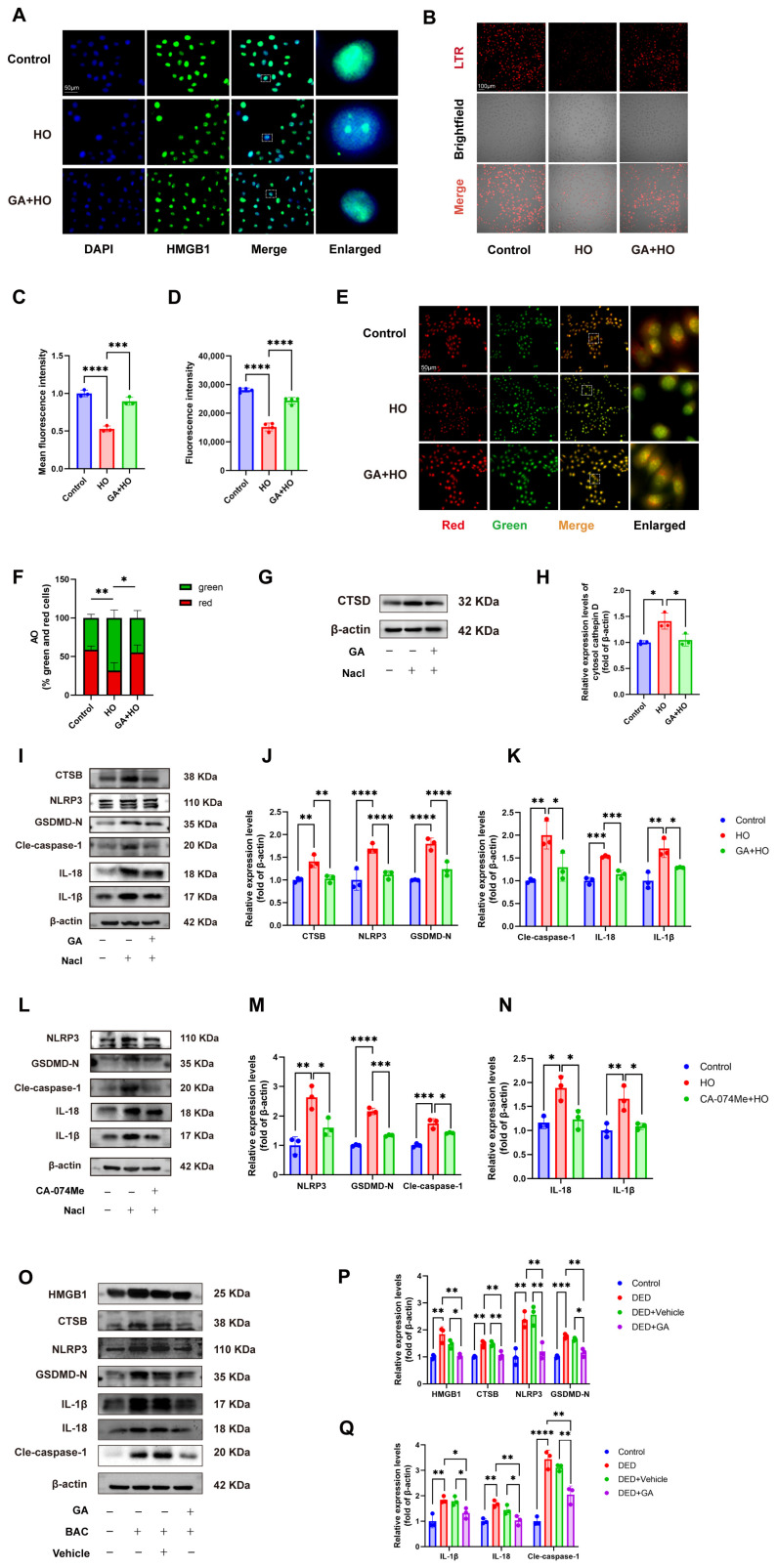
GA downregulates the HMGB1/LMP/CTSB pathway to ameliorate pyroptosis. (**A**) Immunofluorescence staining of HMGB1 nuclear translocation (n = 3, scale bar = 50 μm). (**B**,**C**) Representative fluorescence images (scale bar = 100 μm) and quantified data (n = 3) of LysoTracker Red-stained HCECs. (**D**) Determination of LysoTracker Red fluorescence levels in HCECs from multiple groups using a fluorescence microplate reader (n = 5). (**E**,**F**) Representative fluorescence images (scale bar = 100 μm) and quantified data (n = 3) of AO-labeled HCECs, showing the ratiometric shift between red and green fluorescence. (**G**) HCECs were pretreated with GA for 24 h followed by hyperosmotic stress for 24 h. Western blot analysis of cytosolic CTSD levels. (**H**) Quantification of cytosolic CTSD protein level in vitro (n = 3). (**I**–**K**) HCECs were pretreated with GA for 24 h followed by hyperosmotic stress for 24 h. Western blot analysis of cytosolic CTSB, NLRP3, GSDMD-N, IL-1β, IL-18 and cleaved caspase-1 levels and quantification in vitro (n = 3). (**L**–**N**) HCECs were pretreated with the CTSB inhibitor CA-074Me for 24 h followed by hyperosmotic stress for 24 h. Western blot analysis of NLRP3, GSDMD-N, IL-1β, IL-18 and cleaved caspase-1 levels and quantification in vitro (n = 3). (**O**) Western blot analysis of HMGB1, cytosolic CTSB, NLRP3, GSDMD-N, IL-1β, IL-18 and cleaved caspase-1 levels in different groups, with each sample comprising five corneas. (**P**,**Q**) Quantification of the above protein expressions in vivo (n = 3). Dashed boxes in panels A and E indicate regions selected for magnified views. The data were presented as mean ± SD of at least three independent experiments (* *p* < 0.05, ** *p* < 0.01, *** *p* < 0.001, **** *p* < 0.0001).

GA is a widely known HMGB1 inhibitor [50,51]. We therefore hypothesized that GA might ameliorate DED by modulating this pathway. Although HMGB1 is a secreted protein, total HMGB1 protein levels did not significantly increase in hyperosmotic-treated HCECs by Western blot analysis. However, immunofluorescence staining revealed that hyperosmotic stress induced nuclear-to-cytoplasmic translocation of HMGB1 in HCECs, an effect that was reduced by GA pretreatment (Figure 3A).

We next assessed lysosomal integrity. LysoTracker Red (LTR) staining showed a significant reduction in fluorescence intensity under hyperosmotic stress, indicating impaired lysosomal acidity, whereas GA treatment restored LTR fluorescence (Figure 3B–D). Consistently, acridine orange (AO) staining demonstrated decreased red fluorescence and increased green fluorescence, along with a reduced red/green fluorescence ratio in hyperosmotic-treated HCECs, while GA reversed these changes (Figure 3E,F).

LMP is also characterized by the release of cathepsins (Cathepsin D, B, S, L, etc.) from the lysosome into the cytoplasm, with Cathepsin D (CTSD) being the most abundant. Western blot analysis showed increased cytosolic CTSD levels in hyperosmotic-treated HCECs, which were reduced by GA treatment (Figure 3G,H). Collectively, these results indicate that hyperosmotic stress induces HMGB1 translocation and LMP in HCECs, while GA effectively inhibits this process.

The rupture of the lysosome and the activation and release of lysosomal CTSB are crucial mechanisms for NLRP3 inflammasome activation. In HCECs under hyperosmotic stress, CTSB and pyroptosis-associated mediators (NLRP3, cleaved caspase-1, IL-1β, IL-18) were upregulated, whereas GA pretreatment markedly reduced their expression, indicating attenuation of pyroptosis (Figure 3I–K). Similarly, the CTSB inhibitor CA-074Me decreased pyroptosis protein levels, confirming CTSB as a critical mediator of LMP-induced pyroptosis (Figure 3L–N). Consistent with in vitro findings, Western blot results showed that the expression levels of pyroptosis proteins in the corneas of DED mice were significantly higher compared to the control and vehicle groups, and GA treatment reduced this expression (Figure 3O–Q).

These results collectively demonstrate that GA attenuates pyroptosis in vivo and in vitro by downregulating the HMGB1/LMP/CTSB pathway.

### 2.4. GA Alleviates Hyperosmolarity-Induced Oxidative Stress Damage In Vivo and In Vitro by Ameliorating Mitochondrial Dysfunction and Activating the Nrf2 Signaling Pathway

**Figure 4:**
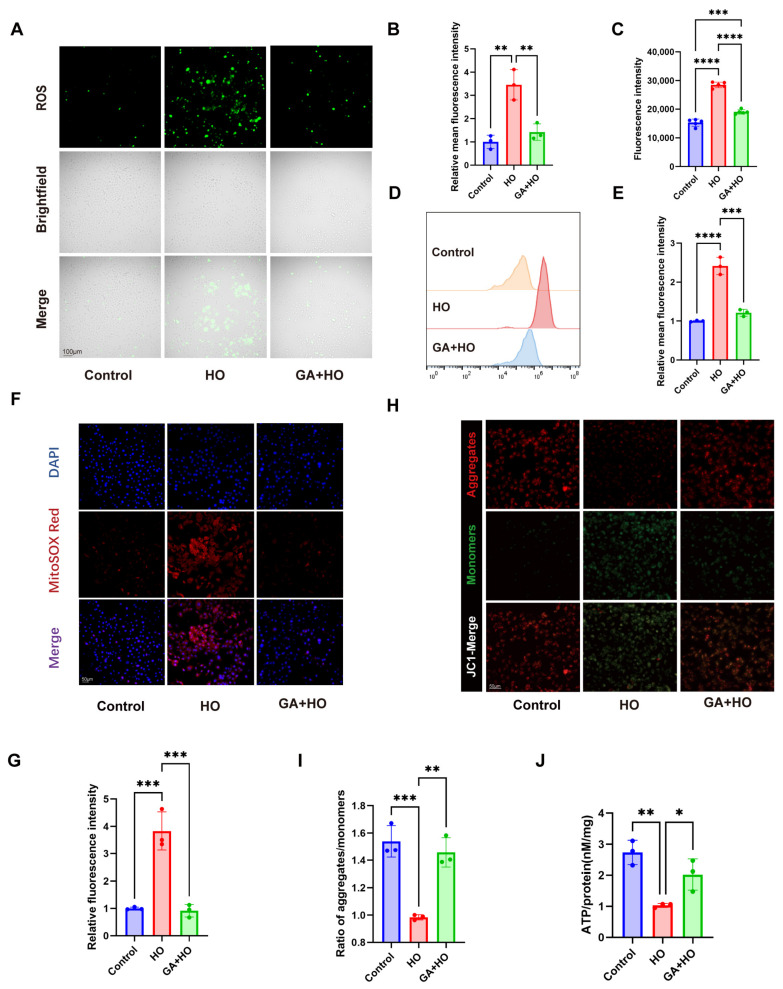
GA alleviates oxidative stress by preserving mitochondrial function. (**A**,**B**) ROS fluorescence in HCECs from different groups observed through fluorescent imaging (scale bar = 100 μm) with subsequent relative intensity analysis (n = 3). (**C**) Quantification of ROS levels measured using a fluorescence microplate reader (excitation/emission monitored around 488 nm; n = 5). (**D**,**E**) ROS fluorescence in HCECs from different groups examined by flow cytometry and quantification of fluorescence intensity (n = 3). (**F**,**G**) MitoSOX Red fluorescence in HCECs from different groups observed through fluorescent imaging (scale bar = 50 μm) with subsequent relative intensity analysis (n = 3). (**H**) JC-1 fluorescence in HCECs from different treatment groups observed through fluorescent imaging (scale bar = 50 μm). (**I**) Quantification of ratio of aggregates/monomers in each group (n = 3). (**J**) Measurement of ATP levels in HCECs (n = 3). The data were presented as mean ± SD of at least three independent experiments (* *p* < 0.05, ** *p* < 0.01, *** *p* < 0.001, **** *p* < 0.0001).

Oxidative stress is a central element in the pathogenesis of DED; thus, we assessed the antioxidant effects of GA. We employed a DCFH-DA probe to detect ROS generation within HCECs. Hyperosmotic insults triggered a surge in intracellular ROS accumulation, an effect that was effectively blunted by GA pretreatment, as evidenced by a decline in fluorescence intensity (Figure 4A,B). Consistently, flow cytometry and microplate analysis confirmed decreased ROS levels in the GA-pretreated group compared with the hyperosmotic group (Figure 4C–E). These outcomes provided evidence for the antioxidant properties of GA.

Given that mitochondria serve as the main source of cellular ROS, we further evaluated mitochondrial function by measuring mitochondrial reactive oxygen species (mtROS), mitochondrial membrane potential (MMP), and adenosine triphosphate (ATP) levels. MitoSOX staining showed that hyperosmotic stress increased mtROS production, which was effectively attenuated by GA pretreatment (Figure 4F,G). JC-1 staining revealed a loss of MMP under hyperosmotic conditions, evidenced by the dissociation of red J-aggregates into green monomers, while GA restored MMP to near-normal levels (Figure 4H,I). In parallel, hyperosmotic stress reduced intracellular ATP levels, whereas GA pretreatment rescued ATP production (Figure 4J).

**Figure 5:**
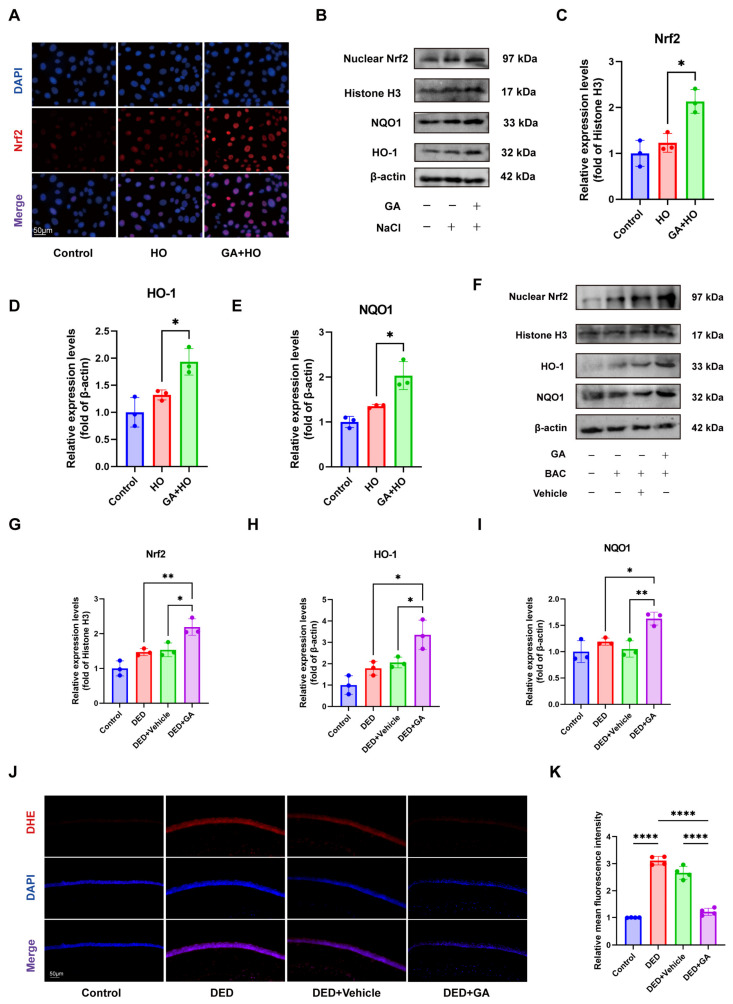
GA activates Nrf2/HO-1/NQO1 signaling pathway to mitigate oxidative stress. (**A**) Nrf2 localization was assessed by immunofluorescence, with DAPI utilized as a nuclear counterstain to demonstrate the nuclear translocation of Nrf2 in HCECs (n = 3, scale bar = 50 μm). (**B**–**E**) HCECs were pretreated with GA for 24 h, followed by hyperosmotic stress for 24 h. Western blot analysis of nuclear Nrf2, HO-1, and NQO1 levels and quantification (n = 3). (**F**–**I**) Western blot analysis of Nrf2 pathway-related proteins, including nuclear Nrf2, HO-1, and NQO1 in different groups, with each sample comprising five corneas and quantification (n = 3). (**J**,**K**) DHE staining of mouse corneal cryosections showing ROS levels and quantification of fluorescence intensity (n = 3, scale bar = 50 μm). The data were presented as mean ± SD of at least three independent experiments (* *p* < 0.05, ** *p* < 0.01, **** *p* < 0.0001).

Nrf2 serves as a pivotal regulator for modulating endogenous antioxidant enzyme levels, which are key protective antioxidants. We therefore investigated whether GA treatment could activate Nrf2. Immunofluorescence staining showed the intracellular location of Nrf2. In the control and hyperosmotic-treated groups, Nrf2 fluorescence was at a low level, which was predominantly confined to the cytoplasm of HCECs. Pretreatment with GA significantly increased Nrf2 expression and activated its nuclear entry (Figure 5A). Western blot analysis further indicated that GA boosted the nuclear accumulation of Nrf2, concurrently elevating the levels of downstream targets heme oxygenase-1 (HO-1) and NAD(P)H quinone dehydrogenase 1 (NQO1), in HCECs (Figure 5B–E).

These findings were consistent in vivo, where GA elevated nuclear Nrf2, HO-1, and NQO1 expression in corneal tissue of DED mice (Figure 5F–I) and reduced ROS levels, as indicated by decreased Dihydroethidium (DHE) fluorescence (Figure 5J,K).

Collectively, these results demonstrate the antioxidative capacity of GA, which is achieved through mitochondrial functional recovery and up-regulation of the Nrf2/HO-1/NQO1 pathway in vivo and in vitro.

**Figure 6:**
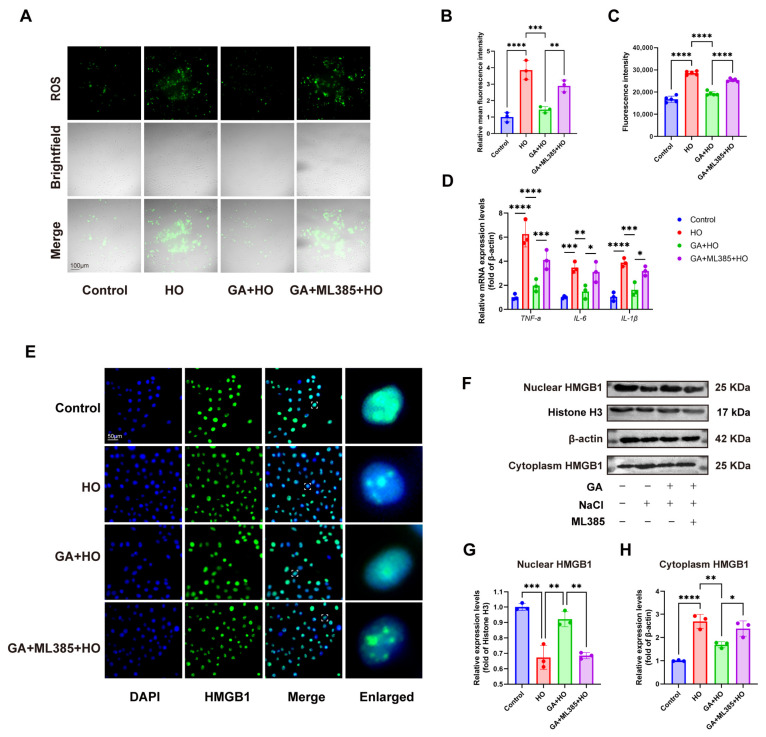
ML385 reverses GA-mediated protection and regulates HMGB1 translocation via Nrf2 inhibition. (**A**,**B**) ROS fluorescence in HCECs from different groups observed through fluorescent imaging (scale bar = 100 μm) with subsequent relative intensity analysis (n = 3). (**C**) Quantification of ROS levels measured using a fluorescence microplate reader (excitation/emission monitored around 488 nm; n = 5). (**D**) RT-qPCR was employed to determine the mRNA levels of key pro-inflammatory cytokines, including *TNF-α*, *IL-1β*, and *IL-6*, within the cells (n = 3). (**E**) Immunofluorescence staining of HMGB1 nuclear translocation (n = 3, scale bar = 50 μm). (**F**–**H**) Nuclear and cytoplasmic fractionation was performed in cells from different treatment groups, followed by Western blot analysis and quantitative densitometry of nuclear and cytoplasmic HMGB1 (n = 3). Dashed boxes in panel E indicate regions selected for magnified views. The data were presented as mean ± SD of at least three independent experiments (* *p* < 0.05, ** *p* < 0.01, *** *p* < 0.001, **** *p* < 0.0001).

To further investigate whether the protective effects of GA involve the Nrf2 signaling pathway, we employed the Nrf2-specific inhibitor ML385 following GA pretreatment. Compared with the GA-treated group, HCECs pretreated with GA and subsequently exposed to ML385 showed significantly increased intracellular ROS levels and elevated expression of inflammatory markers (Figure 6A–D), suggesting that Nrf2 signaling is critically involved in GA-mediated protection.

In addition, ML385 partially reversed the inhibitory effect of GA on HMGB1 nuclear-cytoplasmic translocation (Figure 6E–H), indicating that Nrf2-mediated redox regulation may indirectly influence HMGB1.

### 2.5. GA Protected the Ocular Surface Epithelial Barrier In Vivo and In Vitro by Reducing TNF-α and MMP9

**Figure 7:**
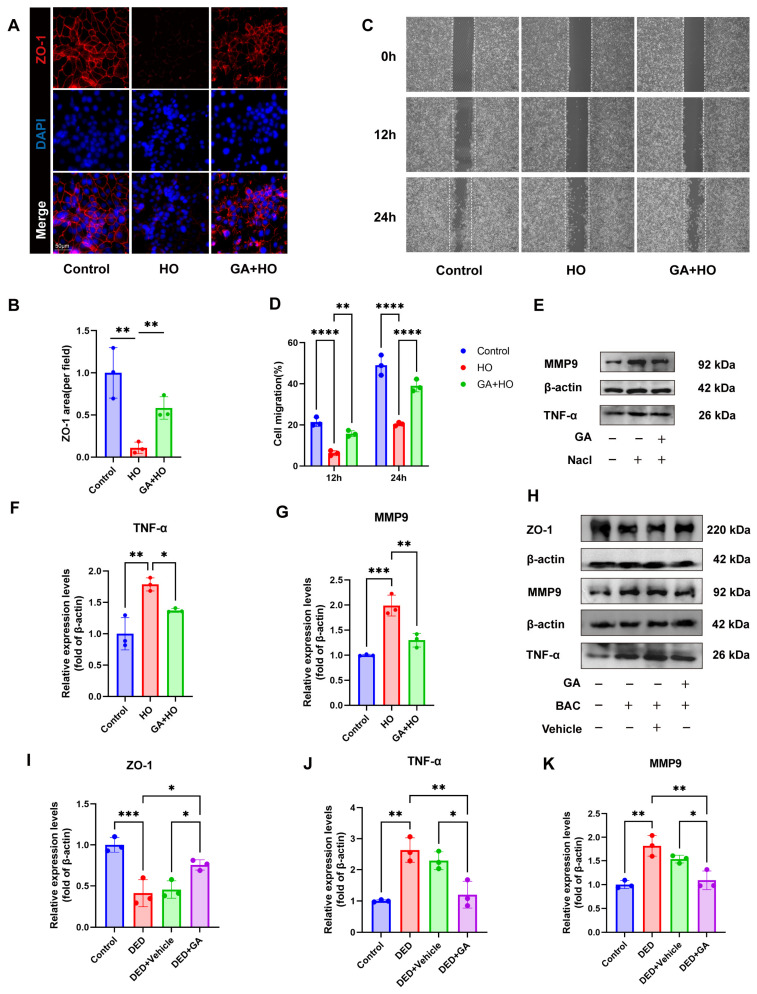
GA protects the epithelial barrier via suppressing TNF-α and MMP9. (**A**) Immunofluorescence staining showing ZO-1 in HCECs (n = 3, scale bar = 50 μm). (**B**) Quantification of ZO-1-positive staining area in vitro (n = 3). (**C**) Wound-healing assays were employed to assess cell migration ability (n = 3, scale bar = 100 μm). (**D**) Measurement of HCECs migration (n = 3). (**E**) HCECs were pretreated with GA for 24 h followed by hyperosmotic stress for 24 h. Western blot analysis of TNF-*α* and MMP9 levels. (**F**,**G**) Quantification of TNF-α and MMP9 expression in vitro (n = 3). (**H**) Western blot analysis of TNF-α and MMP9 in different groups with each sample comprising five corneas. (**I**–**K**) Quantification of TNF-α, MMP9 and ZO-1 expressions in vivo (n = 3). The data were presented as mean ± SD of at least three independent experiments (* *p* < 0.05, ** *p* < 0.01, *** *p* < 0.001, **** *p* < 0.0001).

The corneal epithelial barrier is vital for the maintenance of ocular surface integrity in DED and depends on tight junctions (TJs), with ZO-1 as a key component. Immunofluorescence staining showed that ZO-1 localized continuously in a characteristic reticular pattern at the cell–cell boundaries in normal HCECs, whereas hyperosmotic stress markedly reduced ZO-1-positive areas and disrupted barrier continuity. In comparison, GA pretreatment preserved ZO-1 expression and maintained TJ integrity (Figure 7A,B).

Compromised corneal epithelial barrier function often leads to delayed wound healing. To evaluate the effect of GA on promoting epithelial repair, a scratch wound assay was performed. Hyperosmotic stress significantly impaired HCECs migration at 12 h and 24 h, while GA pretreatment restored migratory capacity (Figure 7C,D).

Given that TNF-α/MMP9 signaling disrupts epithelial barrier integrity through ZO-1 cleavage, we next examined their expression. Western blot analysis showed that hyperosmotic stress increased TNF-α and MMP9 levels, whereas GA pretreatment suppressed their expression (Figure 7E–G). This inhibitory effect was further validated in DED mice. In contrast to the control group, mice in the DED group exhibited markedly elevated corneal expression of both TNF-α and MMP9. Topical ocular pretreatment with GA reversed this increase, significantly lowering their expression (Figure 7H,J,K). In parallel, GA restored ZO-1 expression in vivo (Figure 7I).

Collectively, these consistent in vitro and in vivo findings strongly suggest that GA exerts a potent protective function on the epithelial barrier and promotes corneal epithelial repair by reducing TNF-α and MMP9.

## 3. Discussion

The present research provides a multi-dimensional evaluation of GA’s therapeutic potential in managing DED while uncovering its diverse mechanistic pathways. GA exerted protective effects by inhibiting the HMGB1-LMP-CTSB-pyroptosis pathway, preserving mitochondrial function, reducing oxidative stress, and maintaining corneal epithelial barrier integrity.

HMGB1 is recognized as a crucial Damage-Associated Molecular Pattern (DAMP) and a potent inflammatory driver in the pathology of DED [49,52]. One of the primary receptors for HMGB1 is Toll-like Receptor 4 (TLR4), to which HMGB1 directly binds to regulate immune responses as a chemoattractant or pro-inflammatory mediator [53]. Existing literature has reported that the expression of TLR4 and the levels of its downstream inflammatory cytokines are upregulated in DED [54,55]. Recent research has further highlighted that HMGB1 upregulation in DED compromises lysosomal membrane permeability, leading to LMP [29]. GA treatment suppressed hyperosmolarity-induced HMGB1 translocation, mitigated LMP and CTSB release, and attenuated downstream pyroptosis, highlighting the close pharmacological relevance of GA’s action as an HMGB1 inhibitor.

Oxidative stress is a pivotal contributor to DED pathogenesis, with excessive ROS promoting epithelial apoptosis. Notably, mitochondria are the primary source of intracellular ROS, and mitochondrial dysfunction further amplifies oxidative stress [56]. Our data show that GA preserves mitochondrial membrane potential, restores ATP production, and reduces mtROS levels, indicating that its antioxidant effects may arise from maintaining mitochondrial homeostasis.

Nrf2 serves as a vital regulator for antioxidant enzyme induction. Following its release from cytoplasmic sequestration during stress, Nrf2 occupies the ARE promoter regions to stimulate the expression of HO-1 and NQO1, thereby strengthening the cellular defense against oxidative damage [57,58,59]. GA has been reported to treat ulcerative colitis and myocardial ischemia–reperfusion injury by targeting and stimulating the nuclear import of Nrf2 [60,61]. However, the antioxidative efficacy of GA in DED has not yet been investigated. Our findings extended these observations to DED and suggested that GA exerts its antioxidant effects through a ‘dual-regulatory’ mechanism: upstream, by preserving mitochondrial function to curtail ROS generation at its source, and downstream, by activating Nrf2-dependent antioxidant responses to amplify scavenging capacity. This synergistic modulation of mitochondrial homeostasis and antioxidant signaling pathways likely provides the mechanistic foundation for GA’s efficacy in alleviating oxidative stress-related ocular surface impairment.

Pharmacological inhibition of Nrf2 by ML385 was found to markedly diminish the protective effects of GA, as evidenced by the restoration of ROS levels and inflammatory cytokine expression. These results suggest that Nrf2 activation may be a contributory factor to the therapeutic efficacy of GA in HCECs.

Furthermore, the observation that ML385 treatment partially interfered with GA’s ability to inhibit HMGB1 translocation suggests a potential link between Nrf2 signaling and the subcellular distribution of HMGB1. This finding supports the existence of a functional interplay between these pathways. We speculate that GA-induced Nrf2 signaling may stabilize the intracellular microenvironment, thereby indirectly preventing the oxidative stress-triggered nuclear export of HMGB1. However, the precise mechanisms underlying this interaction remain to be further elucidated.

Epithelial barrier integrity is essential for ocular surface homeostasis, relying on tight and adherens junction proteins such as ZO-1 and E-cadherin. TNF-α and MMP9 contribute to barrier disruption in DED. Previous studies have explored the epithelial barrier protective effects of GA in colorectal-related diseases [62], showing that GA, as a key component of a formulation against ulcerative colitis (UC), markedly upregulated the levels of tight junction markers (ZO-1 and occludin) alongside the adhesion molecule E-cadherin and reduced goblet cell loss [63], thereby repairing intestinal epithelial barrier damage. GA reduced TNF-α and MMP9 expression, preserved ZO-1 levels, and promoted epithelial wound healing, consistent with previous findings on its barrier-protective effects in other diseases.

GA’s broad pharmacological profile, biocompatibility, and long history of dietary use [64,65] underscore its therapeutic potential. Its concurrent anti-inflammatory and antioxidant activities provide advantages over conventional monotherapies. While the current results are compelling, several limitations should be addressed. Firstly, future studies are warranted to extend beyond the ocular surface to evaluate more distant sites, such as the trigeminal ganglia and meibomian/lacrimal glands, which were not comprehensively assessed in our current in vivo model. Given GA’s reported neuroprotective effects on trophic nerves [66,67] a more comprehensive analysis of GA’s impact on neuro-ocular surface function and these accessory ocular tissues represents a significant future research direction. Secondly, the mechanistic findings of this study are primarily based on expression and subcellular localization analyses, which do not fully establish a definitive causal relationship. Although pharmacological inhibition of Nrf2 provided supportive functional evidence, more rigorous approaches, such as genetic or rescue experiments, are required to further validate the causal links between Nrf2 activation and HMGB1 regulation. Thirdly, only a BAC-induced DED model was used; employing additional established models could improve the reliability and generalizability of our findings. Finally, GA was administered as a simple solvent-based eye drop. Considering its ability to self-assemble into micelles, inclusion complexes, nanofibers, and hydrogels, which enhance solubility, stability, and membrane permeability [68], developing GA-based delivery systems may prolong ocular retention and improve bioavailability and therapeutic efficacy.

Collectively, these findings suggest that GA emerges as a compelling therapeutic option for DED, offering mechanistic insights and a foundation for future translational application.

## 4. Materials and Methods

### 4.1. Animals, Benzalkonium Chloride (BAC)-Induced DED Model, and Clinical Assessments

This research followed ARVO principles and was conducted under the ethical oversight of the Nanjing Drum Tower Hospital Animal Research Committee (Approval No. 2023AE01042, 28 November 2023). Based on the effective in vitro concentration (200 μM), and considering the low ocular bioavailability of eye drops (approximately 5%) [69,70,71] due to tear turnover and nasolacrimal drainage, the in vivo dose was scaled up to ensure sufficient ocular surface exposure. Female C57BL/6 mice (6–8 weeks) were randomly assigned to five groups: Control, DED (0.075% BAC for 1 week), Vehicle (DED + 0.1% DMSO), GA treatment (DED + 4 mM GA in 0.1% DMSO), and Sodium Hyaluronate (DED + SH). Following the initial one-week induction period of DED, the DED group continued to receive BAC, while the treatment groups received 5 μL eye drops twice daily, with at least 3 h between modeling and treatment. An additional safety evaluation was performed in parallel, and the corresponding results have been included in the Supplementary Materials. To evaluate DED progression, tear production and corneal epithelial defects were monitored on days 0, 7, and 14 using phenol red cotton threads and 0.25% fluorescein staining, respectively. Mice were euthanized at the study endpoint for sample collection.

### 4.2. Periodic Acid Schiff (PAS) and Hematoxylin and Eosin (H&E) Staining

Following fixation in 4% PFA, corneal samples were embedded into paraffin blocks. Subsequently, a microtome was used to obtain 5 µm-thick sections for histological analysis. Sections were cleared in xylene and stained with H&E using a commercial kit (Servicebio, Wuhan, China), then dehydrated, cleared, and mounted. Goblet cell density was assessed by PAS staining (Solarbio, Beijing, China). Representative images were captured with a light microscope (Leica Microsystems, Wetzlar, Germany).

### 4.3. Cell Culture and Treatment

Human corneal epithelial cells (HCECs; CRL-11135, ATCC, Manassas, VA, USA) were cultured in DMEM/F12 supplemented with 10% FBS and 1% penicillin/streptomycin in a humidified 37 °C, 5% CO_2_ incubator. To model hyperosmotic stress in vitro, 90 mM NaCl was added to the medium [40,72,73]. For GA treatment, cells were pretreated with 200 μM GA (≥98%, MedChemExpress, Shanghai, China) for 24 h prior to hyperosmotic exposure. ML385 (1 μM) [74,75], a selective inhibitor of Nrf2, was applied for 2 h following GA pretreatment to pharmacologically suppress Nrf2 signaling.

### 4.4. Assessment of Cytotoxicity and Cytoprotective Effects

For the 24 h treatment assays, HCECs (4 × 10^3^ cells/well) were partitioned into 96-well plates and administered various levels of GA. The viability of HCECs was assessed through the CCK-8 assay (10 μL/well). The absorbance values were subsequently captured at 450 nm after 1–2 h. For rescue experiments, cells were pretreated with GA and then exposed to hyperosmotic medium before the CCK-8 assay.

Total RNA was extracted from HCECs using FreeZol (Vazyme, Nanjing, China), and 1 μg underwent reverse transcription into cDNA via the HiScript gDNA Removal RT MasterMix (CWBIO, Beijing, China). The resulting cDNA was amplified using MagicSYBR Mixture (CWBIO, China) on the QuantStudio 6 Flex real-time PCR platform (Invitrogen, Carlsbad, CA, USA). Relative mRNA levels were assessed using the 2^−ΔΔCT^ method [76,77], with β-actin serving as the internal standard for all quantitative analyses. Primer sequences are listed in Table 1.

### 4.5. Analysis of Cell Apoptosis Rate

Following cell harvest, HCECs underwent co-incubation with FITC-conjugated Annexin V and Propidium Iodide (PI) in an environment protected from light using an Apoptosis Detection Kit (KeyGEN BioTECH, Nanjing, China). Early and late apoptosis were measured using the Accuri C6 platform (BD, Franklin Lakes, NJ, USA). Data analysis was carried out via FlowJo V10 software (FlowJo, Ashland, OR, USA).

### 4.6. Immunofluorescence Staining

After the stabilization (4% PFA, 30 min) and permeabilization (0.5% Triton X-100, 1 h) of HCECs treated with GA and hyperosmotic medium, a blocking step with 5% BSA for 30 min at room temperature was conducted. After washing, primary antibodies against Nrf2 (Proteintech, Wuhan, China) and ZO-1 (Huabio, Hangzhou, China) were applied overnight at 4 °C. Cells were then treated with Alexa Fluor 568 secondary antibody (1 h) and DAPI (5 min) for nuclear staining, with final imaging performed on a Leica THUNDER system.

### 4.7. Assessment of Lysosomal Membrane Permeabilization (LMP)

HCECs were stained with LysoTracker Red (Beyotime, Shanghai, China) or Acridine Orange (5 μM, Solarbio, Beijing, China) at 37 °C for 30 min, followed by three PBS washes. Fluorescence was imaged using a Leica THUNDER system and quantified via flow cytometry (Accuri C6) and microplate reader (Tecan SPARK, Männedorf, Switzerland). LTR: Ex/Em 577/590 nm; AO: green (488/530 nm) for cytosolic/nuclear signal, red (488/640 nm) for lysosomes. LMP was assessed by the red-to-green fluorescence ratio.

### 4.8. Western Blot Analysis

Total protein extraction was carried out with RIPA buffer (CWBIO, Beijing, China) with 10 min lysis, followed by centrifugation at 13,000 rpm. Nuclear and cytoplasmic fractions for Nrf2 analysis were separated using a Nuclear/Cytosol Protein Extraction Kit (Beyotime, Shanghai, China).

Cytoplasmic CTSD and CTSB were isolated free of lysosomal contamination using a Lysosome Protein Extraction Kit (Solarbio, Beijing, China). HCECs were resuspended in Extraction Reagent A, shaken on ice for 10 min, homogenized (40 strokes), and centrifuged at 1000× *g* for 5 min, then at 30,000× *g* for 30 min. The final supernatant was collected as the cytoplasmic fraction.

Standard BCA protocols (CWBIO, Beijing, China) were employed for protein measurement, with subsequent SDS-PAGE electrophoresis (FDbio, Hangzhou, China) and PVDF membrane blotting (Millipore, Billerica, MA, USA) performed according to established Western blot procedures. Primary antibodies included HMGB1, CTSD, CTSB, IL-18 (Abways, Shanghai, China), IL-1β (Abmart, Shanghai, China), cleaved caspase-1, NLRP3, Nrf2, TNF-α (Proteintech, Wuhan, China), NQO1, HO-1 (Immunoway, Shanghai, China), ZO-1 (Huabio, Hangzhou, China), and MMP9 (Wanleibio, Shenyang, China).

After primary antibody incubation, membranes were washed with TBST and incubated with HRP-conjugated secondary antibodies (FDbio, Hangzhou, China, 1:10,000). Protein bands were visualized using ECL reagents (Vazyme, Nanjing, China) and quantified with ImageJ software (version 1.53e, NIH, USA).

### 4.9. Measurement of Intracellular ROS Level

Intracellular ROS generation in HCECs was assessed via a DCFH-DA assay kit (Beyotime, Shanghai, China). Cells were stained for 30 min at 37 °C and washed with PBS. Fluorescence was measured using a Leica THUNDER microscope, Accuri C6 flow cytometer (BD, Franklin Lakes, NJ, USA), and Tecan SPARK microplate reader. Data were analyzed with ImageJ and FlowJo V10.

### 4.10. Measurement of Corneal Oxidative Stress

Fresh ocular tissues were embedded in OCT (Tissue-Tek^®^, SAKURA, Torrance, CA, USA) and cryosectioned. Following a 20 min incubation with 10 μM DHE (TargetMol, Shanghai, China) at 37 °C, the sections underwent triple rinses in TBST buffer. Slides were mounted with DAPI-containing antifade medium and covered. Fluorescence was imaged using a Leica THUNDER Imager (Leica, Wetzlar, Germany) with 535 nm excitation and 610 nm emission.

### 4.11. Assessment of Mitochondrial Oxidative Stress and Function

mtROS in HCECs was detected using 5 μM MitoSOX Red (Beyotime, Shanghai, China) for 30 min at 37 °C, followed by three PBS washes and mounting with DAPI-containing antifade medium. JC-1 (Beyotime, Shanghai, China) was employed for mitochondrial potential assessment. After an incubation interval of 15–20 min at 37 °C, cells were washed and then imaged for green (490/530 nm) and red (525/590 nm) fluorescence. The resulting red/green fluorescence ratio was calculated to reflect MMP. Cellular ATP was measured in lysates (1 × 10^4^ cells/well) using a bioluminescence assay kit (Beyotime, Shanghai, China).

### 4.12. Scratch Wound Healing Assay

HCECs were seeded at 1 × 10^5^ cells/well in 6-well plates with 2 mL complete medium and cultured overnight to form a confluent monolayer. A consistent scratch was generated using a 200-μL sterile tip, after which prewarmed PBS rinses were repeated 2–3 times to ensure the removal of dislodged cells. Finally, 2 mL of medium without serum was replenished in each well. Cells in the GA group were pretreated with 200 μM GA for 24 h, then replaced with 90 mM NaCl for the scratch assay. Hyperosmotic-treated group cells were directly exposed to hyperosmotic medium without GA pretreatment. Images of three representative fields per well were captured at 0, 12, and 24 h using an inverted microscope under consistent magnification and focus. The scratch area was measured with ImageJ, and the migration rate was calculated as: Migration rate (%) = (Area_0h_ − Area_12/24h_)/Area_0h_ × 100%.

### 4.13. Statistical Analysis

Statistical analyses were executed using GraphPad Prism 9.0, with data expressed as mean ± SD derived from no fewer than three independent trials. The significance of differences was assessed by unpaired Student’s *t*-tests for pairwise comparisons, while one-way or two-way ANOVA was utilized for evaluating multiple groups. Differences were recognized as statistically significant at *p* < 0.05.

## 5. Conclusions

**Figure 8:**
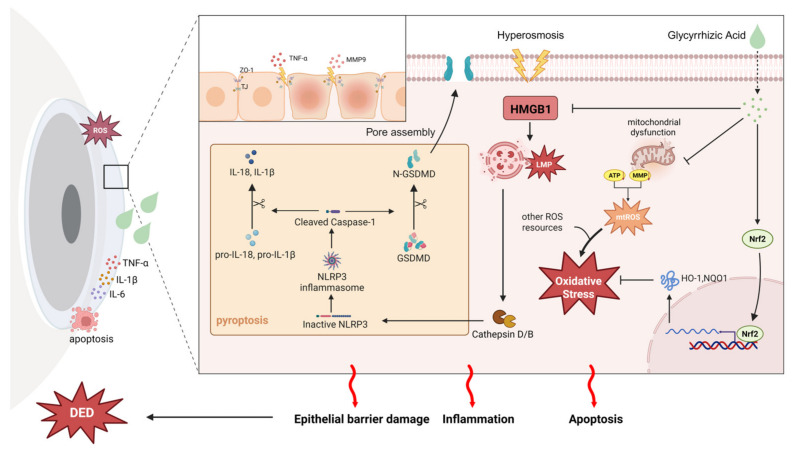
Schematic illustration of the multifaceted protective mechanisms of GA in DED. Hyperosmotic stress activates HMGB1, which induces lysosomal membrane permeabilization to promote pyroptosis via cathepsin B. This stress also impairs mitochondrial function, resulting in robust ROS production, and elevates TNF-α and MMP9 levels to disrupt ZO-1 tight junctions. GA effectively counteracts these pathological processes by: (1) inhibiting the HMGB1/LMP/CTSB pathway to suppress pyroptosis; (2) activating the Nrf2/HO-1/NQO1 antioxidant pathway to restore mitochondrial homeostasis and neutralize ROS; and (3) downregulating the TNF-*α*/MMP9 pathway to preserve the ocular surface epithelial barrier. Collectively, GA protects the ocular surface from oxidative damage, chronic inflammation, and barrier dysfunction.

In summary, our results provide compelling evidence that GA may serve as a promising candidate for treating DED through its multi-target mechanisms. GA effectively alleviates hyperosmolarity-induced inflammation and corneal damage by modulating the HMGB1/LMP/CTSB/pyroptosis pathway, preserving mitochondrial function, reducing oxidative stress, and maintaining epithelial barrier integrity. These findings reveal the complex interplay of molecular mechanisms underlying DED (Figure 8) and suggest that GA could be further developed as a therapeutic agent. Future studies are needed to clarify its precise molecular targets, evaluate clinical efficacy, and optimize delivery strategies for effective treatment.

## Figures and Tables

**Table 1:** Primers used for RT-qPCR.

Gene	Forward Primer	Reverse Primer
*TNF-α* (Human)	GTTCCCCAGGGACCTCTCTC	GGCTACAGGCTTGTCACTCG
*IL-6* (Human)	CCAGAGCTGTGCAGATGAGT	ATTTGTGGTTGGGTCAGGGG
*IL-1β* (Human)	AAGCAGCCATGGCAGAAGTA	GGTGGTCGGAGATTCGTAGC
*β-actin* (Human)	ACAGAGCCTCGCCTTTGC	GCGGCGATATCATCATCC
*TNF-α* (Mouse)	CCCTCACACTCAGATCATCTTCT	GCTACGACGTGGGCTACAG
*IL-6* (Mouse)	TAGTCCTTCCTACCCCAATTTCC	TTGGTCCTTAGCCACTCCTTC
*IL-1β* (Mouse)	GAAATGCCACCTTTTGACAGTG	TGGATGCTCTCATCAGGACAG
*β-actin* (Mouse)	GGCTGTATTCCCCTCCATCG	CCAGTTGGTAACAATGCCATGT

## Data Availability

The original contributions presented in this study are included in the article/Supplementary Materials. Further inquiries can be directed to the corresponding author.

## Supplementary Materials

The following supporting information can be downloaded at: https://www.mdpi.com/article/10.3390/ijms27094153/s1.

## Abbreviations

The following abbreviations are used in this manuscript:

DED	Dry Eye Disease
GA	Glycyrrhizic Acid
SOD	Superoxide Dismutase
GPX	Glutathione Peroxidase
IL-1β	Interleukin-1β
TNF-α	Tumor Necrosis Factor-α
MMP9	Matrix Metalloprotease 9
HMGB1	High Mobility Group Box 1
HCECs	Human Corneal Epithelial Cells
BAC	Benzalkonium Chloride
SH	Sodium Hyaluronate
H&E	Hematoxylin and Eosin
PAS	Periodic Acid–Schiff
ZO-1	Zonula Occludens-1
TJs	Tight Junctions
mtROS	Mitochondrial Reactive Oxygen Species
MMP	Mitochondrial Membrane Potential
ATP	Adenosine Triphosphate
Nrf2	Nuclear Factor Erythroid 2–Related Factor 2
HO-1	Heme Oxygenase-1
NQO1	NAD (P)H Quinone Dehydrogenase 1
LMP	Lysosomal Membrane Permeabilization
CTSB	Cathepsin B
CTSD	Cathepsin D
LTR	LysoTracker Red
AO	Acridine Orange
DHE	Dihydroethidium
LFA-1	Lymphocyte Function-associated Antigen 1

## References

[1] Craig J.P., Nichols K.K., Akpek E.K., Caffery B., Dua H.S., Joo C.K., Liu Z., Nelson J.D., Nichols J.J., Tsubota K. (2017). TFOS DEWS II Definition and Classification Report. Ocul. Surf..

[2] Zemanová M. (2021). Dry Eye Disease. A Review. Ceska Slov. Oftalmol..

[3] Clayton J.A. (2018). Dry eye. N. Engl. J. Med..

[4] Stapleton F., Argüeso P., Asbell P., Azar D., Bosworth C., Chen W., Ciolino J.B., Craig J.P., Gallar J., Galor A. (2025). TFOS DEWS III: Digest. Am. J. Ophthalmol..

[5] Xiao K., Li L., Zhang X., Ye Y., Yao Y., Liu Y., Chen W., Wang X., Gu C., He M. (2025). Global prevalence of dry Eye: A systematic review and meta-analysis. Cont. Lens Anterior Eye.

[6] Ismail M.F., Khalafalla I., Qarbote A.I. (2025). Prevalence of dry eye disease in African populations: A systematic review and meta-analysis. BMC Ophthalmol..

[7] Zou Y., Li D., Gianni V., Congdon N., Piyasena P., Prakalapakorn S.G., Zhang R., Zhao Z., Chan V.F., Yu M. (2025). Prevalence of dry eye disease among children: A systematic review and meta-analysis. BMJ Open Ophthalmol..

[8] Benítez-Del-Castillo J.M., Burgos-Blasco B. (2025). Prevalence of dry eye disease in Spain: A population-based survey (PrevEOS). Ocul. Surf..

[9] Gomes J.A.P., Santo R.M. (2019). The impact of dry eye disease treatment on patient satisfaction and quality of life: A review. Ocul. Surf..

[10] Barabino S., Labetoulle M., Rolando M., Messmer E.M. (2016). Understanding Symptoms and Quality of Life in Patients with Dry Eye Syndrome. Ocul. Surf..

[11] Kai J.Y., Wu Y.B., Shi B., Li D.L., Dong X.X., Wang P., Pan C.W. (2024). Dry eye symptoms and health-related quality of life among Chinese individuals: A national-based study. Br. J. Ophthalmol..

[12] Villani E., Barabino S., Giannaccare G., Di Zazzo A., Aragona P., Rolando M. (2025). From Symptoms to Satisfaction: Optimizing Patient-Centered Care in Dry Eye Disease. J. Clin. Med..

[13] van Tilborg M.M., Murphy P.J., Evans K.S. (2017). Impact of Dry Eye Symptoms and Daily Activities in a Modern Office. Optom. Vis. Sci..

[14] Nichols K.K., Bacharach J., Holland E., Kislan T., Shettle L., Lunacsek O., Lennert B., Burk C., Patel V. (2016). Impact of Dry Eye Disease on Work Productivity, and Patients’ Satisfaction with Over-the-Counter Dry Eye Treatments. Investig. Ophthalmol. Vis. Sci..

[15] Di Zazzo A., De Gregorio C., Spelta S., Demircan S. (2025). Mental burden of ocular surface discomfort. Eur. J. Ophthalmol..

[16] Wan K.H., Chen L.J., Young A.L. (2016). Depression and anxiety in dry eye disease: A systematic review and meta-analysis. Eye.

[17] Scarpellini C., Ramos Llorca A., Lanthier C., Klejborowska G., Augustyns K. (2023). The Potential Role of Regulated Cell Death in Dry Eye Diseases and Ocular Surface Dysfunction. Int. J. Mol. Sci..

[18] Jeyabalan N., Pillai A.M., Khamar P., Shetty R., Mohan R.R., Ghosh A. (2023). Autophagy in dry eye disease: Therapeutic implications of autophagy modulators on the ocular surface. Indian J. Ophthalmol..

[19] Chen X., Hong J., Le Q. (2025). Role of mitochondrial dysfunction in ocular surface diseases. Cell Stress.

[20] Liu S.H., Saldanha I.J., Abraham A.G., Rittiphairoj T., Hauswirth S., Gregory D., Ifantides C., Li T. (2022). Topical corticosteroids for dry eye. Cochrane Database Syst. Rev..

[21] Wan K.H., Chen L.J., Young A.L. (2015). Efficacy and Safety of Topical 0.05% Cyclosporine Eye Drops in the Treatment of Dry Eye Syndrome: A Systematic Review and Meta-analysis. Ocul. Surf..

[22] de Paiva C.S., Pflugfelder S.C., Ng S.M., Akpek E.K. (2019). Topical cyclosporine A therapy for dry eye syndrome. Cochrane Database Syst. Rev..

[23] Keating G.M. (2017). Lifitegrast Ophthalmic Solution 5%: A Review in Dry Eye Disease. Drugs.

[24] Cui D., Saldanha I.J., Li G., Mathews P.M., Lin M.X., Akpek E.K. (2024). United States Regulatory Approval of Topical Treatments for Dry Eye. Am. J. Ophthalmol..

[25] Messmer E.M. (2015). The pathophysiology, diagnosis, and treatment of dry eye disease. Dtsch. Arztebl. Int..

[26] Boya P., Kroemer G. (2008). Lysosomal membrane permeabilization in cell death. Oncogene.

[27] Feng L., Liang L., Zhang S., Yang J., Yue Y., Zhang X. (2022). HMGB1 downregulation in retinal pigment epithelial cells protects against diabetic retinopathy through the autophagy-lysosome pathway. Autophagy.

[28] Liu S., Perez P., Sun X., Chen K., Fatirkhorani R., Mammadova J., Wang Z. (2024). MLKL polymerization-induced lysosomal membrane permeabilization promotes necroptosis. Cell Death Differ..

[29] Hu X., Feng J., Pan C., Sun Z., Liu J., Xie S., Xiao D., Ma X., Zheng Q., Chen W. (2025). HMGB1 Promotes Lysosome-Dependent Cell Death Induced Via Dry Eye by Disrupting Lysosomal Homeostasis. Investig. Ophthalmol. Vis. Sci..

[30] Bu J., Liu Y., Zhang R., Lin S., Zhuang J., Sun L., Zhang L., He H., Zong R., Wu Y. (2024). Potential New Target for Dry Eye Disease—Oxidative Stress. Antioxidants.

[31] Dammak A., Pastrana C., Martin-Gil A., Carpena-Torres C., Peral Cerda A., Simovart M., Alarma P., Huete-Toral F., Carracedo G. (2023). Oxidative Stress in the Anterior Ocular Diseases: Diagnostic and Treatment. Biomedicines.

[32] Deng R., Hua X., Li J., Chi W., Zhang Z., Lu F., Zhang L., Pflugfelder S.C., Li D.Q. (2015). Oxidative stress markers induced by hyperosmolarity in primary human corneal epithelial cells. PLoS ONE.

[33] Seen S., Tong L. (2018). Dry eye disease and oxidative stress. Acta Ophthalmol..

[34] Liu H., Gambino F., Algenio C.S., Wu C., Gao Y., Bouchard C.S., Qiao L., Bu P., Zhao S. (2020). Inflammation and oxidative stress induced by lipid peroxidation metabolite 4-hydroxynonenal in human corneal epithelial cells. Graefes Arch. Clin. Exp. Ophthalmol..

[35] Park B., Jo K., Lee T.G., Hyun S.W., Kim J.S., Kim C.S. (2019). Polydatin Inhibits NLRP3 Inflammasome in Dry Eye Disease by Attenuating Oxidative Stress and Inhibiting the NF-κB Pathway. Nutrients.

[36] Blaser H., Dostert C., Mak T.W., Brenner D. (2016). TNF and ROS Crosstalk in Inflammation. Trends Cell Biol..

[37] Rhee M.K., Mah F.S. (2017). Inflammation in Dry Eye Disease: How Do We Break the Cycle?. Ophthalmology.

[38] Hybertson B.M., Gao B., Bose S.K., McCord J.M. (2011). Oxidative stress in health and disease: The therapeutic potential of Nrf2 activation. Mol. Asp. Med..

[39] Loboda A., Damulewicz M., Pyza E., Jozkowicz A., Dulak J. (2016). Role of Nrf2/HO-1 system in development, oxidative stress response and diseases: An evolutionarily conserved mechanism. Cell. Mol. Life Sci..

[40] Liang Q., Guo R., Tsao J.R., He Y., Wang C., Jiang J., Zhang D., Chen T., Yue T., Hu K. (2023). Salidroside alleviates oxidative stress in dry eye disease by activating autophagy through AMPK-Sirt1 pathway. Int. Immunopharmacol..

[41] Chen T., Zhou N., Liang Q., Li Q., Li B., Chu Y., Zhang D., Chen Z., Tsao J.R., Feng X. (2024). Biochanin A: Disrupting the inflammatory vicious cycle for dry eye disease. Eur. J. Pharmacol..

[42] Zheng X., Wu Y., Feng Y., Cai M., Zhang M., Zhang L., Yang D., Mao T., Gu H., Ou S. (2026). The aging cornea: From mechanisms to clinical applications. Ageing Res. Rev..

[43] Wesemann D.R., Nagler C.R. (2016). The Microbiome, Timing, and Barrier Function in the Context of Allergic Disease. Immunity.

[44] Suzuki K., Saito J., Yanai R., Yamada N., Chikama T., Seki K., Nishida T. (2003). Cell-matrix and cell-cell interactions during corneal epithelial wound healing. Prog. Retin. Eye Res..

[45] Zhang Y., Yang M., Zhao S.X., Nie L., Shen L.J., Han W. (2022). Hyperosmolarity disrupts tight junction via TNF-α/MMP pathway in primary human corneal epithelial cells. Int. J. Ophthalmol..

[46] Pflugfelder S.C., Farley W., Luo L., Chen L.Z., de Paiva C.S., Olmos L.C., Li D.Q., Fini M.E. (2005). Matrix metalloproteinase-9 knockout confers resistance to corneal epithelial barrier disruption in experimental dry eye. Am. J. Pathol..

[47] Jiang M., Zhao S., Yang S., Lin X., He X., Wei X., Song Q., Li R., Fu C., Zhang J. (2020). An “essential herbal medicine”-licorice: A review of phytochemicals and its effects in combination preparations. J. Ethnopharmacol..

[48] Cinatl J., Morgenstern B., Bauer G., Chandra P., Rabenau H., Doerr H.W. (2003). Glycyrrhizin, an active component of liquorice roots, and replication of SARS-associated coronavirus. Lancet.

[49] Burillon C., Chiambaretta F., Pisella P.J. (2018). Efficacy and safety of glycyrrhizin 2.5% eye drops in the treatment of moderate dry eye disease: Results from a prospective, open-label pilot study. Clin. Ophthalmol..

[50] Yu J., Qu H., Xu W., Fang Y., Wang N., Shi W. (2026). Mechanisms and therapeutic potential of glycyrrhizic acid: Insights into key signaling pathways and disease modulation (Review). Mol. Med. Rep..

[51] Mollica L., De Marchis F., Spitaleri A., Dallacosta C., Pennacchini D., Zamai M., Agresti A., Trisciuoglio L., Musco G., Bianchi M.E. (2007). Glycyrrhizin binds to high-mobility group box 1 protein and inhibits its cytokine activities. Chem. Biol..

[52] Lema C., Reins R.Y., Redfern R.L. (2018). High-Mobility Group Box 1 in Dry Eye Inflammation. Investig. Ophthalmol. Vis. Sci..

[53] Yang H., Wang H., Andersson U. (2020). Targeting Inflammation Driven by HMGB1. Front. Immunol..

[54] Wang Y., Xu Z., Wei L., Lu Y., Shi Y., Wen S., Lv X., Huang K., Lu F., Qu J. (2025). KGF-2 Alleviates Dry Eye Disease by Regulating the HMGB1/TLR4 Pathway. Investig. Ophthalmol. Vis. Sci..

[55] Shen J., Liang Y., Bi Z., Yin X., Chen C., Zhao X., Liu S., Li Y. (2023). Cyclosporin A improves the hyperosmotic response in an experimental dry eye model by inhibiting the HMGB1/TLR4/NF-κB signaling pathway. Exp. Eye Res..

[56] Xu X., Pang Y., Fan X. (2025). Mitochondria in oxidative stress, inflammation and aging: From mechanisms to therapeutic advances. Signal Transduct. Target. Ther..

[57] Hsieh C.Y., Hsiao H.Y., Wu W.Y., Liu C.A., Tsai Y.C., Chao Y.J., Wang D.L., Hsieh H.J. (2009). Regulation of shear-induced nuclear translocation of the Nrf2 transcription factor in endothelial cells. J. Biomed. Sci..

[58] Xu L., He S., Yin P., Li D., Mei C., Yu X., Shi Y., Jiang L., Liu F. (2016). Punicalagin induces Nrf2 translocation and HO-1 expression via PI3K/Akt, protecting rat intestinal epithelial cells from oxidative stress. Int. J. Hyperth..

[59] Xu C., Yuan X., Pan Z., Shen G., Kim J.H., Yu S., Khor T.O., Li W., Ma J., Kong A.N. (2006). Mechanism of action of isothiocyanates: The induction of ARE-regulated genes is associated with activation of ERK and JNK and the phosphorylation and nuclear translocation of Nrf2. Mol. Cancer Ther..

[60] Wang Y., Sun C., Cao Y., Jiao T., Wang K., Li J., Zhang M., Jiang J., Zhong X., Yu S. (2025). Glycyrrhizic acid and patchouli alcohol in Huoxiang Zhengqi attenuate intestinal inflammation and barrier injury via regulating endogenous corticosterone metabolism mediated by 11β-HSD1. J. Ethnopharmacol..

[61] Xu C., Liang C., Sun W., Chen J., Chen X. (2018). Glycyrrhizic acid ameliorates myocardial ischemic injury by the regulation of inflammation and oxidative state. Drug Des. Dev. Ther..

[62] Leong Y.Y., Tong L. (2015). Barrier function in the ocular surface: From conventional paradigms to new opportunities. Ocul. Surf..

[63] Kong J., Xiang Q., Ge W., Wang Y., Xu F., Shi G. (2024). Network pharmacology mechanisms and experimental verification of licorice in the treatment of ulcerative colitis. J. Ethnopharmacol..

[64] Liu J., Banuvar S., Viana M., Barengolts E., Chen S.N., Pauli G.F., van Breemen R.B. (2023). Pharmacokinetic Interactions of a Licorice Dietary Supplement with Cytochrome P450 Enzymes in Female Participants. Drug Metab. Dispos..

[65] Cheng H.S., Yaw H.P., Ton S.H., Choy S.M., Kong J.M., Abdul Kadir K. (2016). Glycyrrhizic acid prevents high calorie diet-induced metabolic aberrations despite the suppression of peroxisome proliferator-activated receptor γ expression. Nutrition.

[66] Kawada K., Ishida T., Jobu K., Morisawa S., Nishida M., Tamura N., Yoshioka S., Miyamura M. (2023). Glycyrrhizae Radix suppresses lipopolysaccharide- and diazepam-induced nerve inflammation in the hippocampus, and contracts the duration of pentobarbital- induced loss of righting reflex in a mouse model. J. Nat. Med..

[67] Yue Y., Wang J., Tian J. (2024). Glycyrrhizic acid promote remyelination after peripheral nerve injury by reducing NF-κB activation. Neurosci. Lett..

[68] Trindade S.G., Okasaki F.B., Williams A.P., Sabadini E., Lutz-Bueno V. (2025). The self-assembly of glycyrrhizic acid into nanofibrils. J. Colloid Interface Sci..

[69] Yang Y., Lockwood A. (2022). Topical ocular drug delivery systems: Innovations for an unmet need. Exp. Eye Res..

[70] Mazet R., Yaméogo J.B.G., Wouessidjewe D., Choisnard L., Gèze A. (2020). Recent Advances in the Design of Topical Ophthalmic Delivery Systems in the Treatment of Ocular Surface Inflammation and Their Biopharmaceutical Evaluation. Pharmaceutics.

[71] Fayyaz A., Ranta V.P., Toropainen E., Vellonen K.S., Valtari A., Puranen J., Ruponen M., Gardner I., Urtti A., Jamei M. (2020). Topical ocular pharmacokinetics and bioavailability for a cocktail of atenolol, timolol and betaxolol in rabbits. Eur. J. Pharm. Sci..

[72] Li B., Liu J., Zhang D., Chu Y., Chen Z., Tsao J., Chen T., Jiang J., Hu K. (2025). Evodiamine Promotes Autophagy and Alleviates Oxidative Stress in Dry Eye Disease Through the p53/mTOR Pathway. Investig. Ophthalmol. Vis. Sci..

[73] Zhang D., Liang Q., Jiang J., Liu W., Chu Y., Chen Z., Li B., Chen T., Tsao J.R., Hu K. (2025). SIRT3 mitigates dry eye disease through the activation of autophagy by deacetylation of FOXO1. Exp. Eye Res..

[74] Yuan Y., Zhai Y., Chen J., Xu X., Wang H. (2021). Kaempferol Ameliorates Oxygen-Glucose Deprivation/Reoxygenation-Induced Neuronal Ferroptosis by Activating Nrf2/SLC7A11/GPX4 Axis. Biomolecules.

[75] Zhao L., Yue Z., Wang G., Qin J., Ma H., Tang D., Yin G. (2025). Smilax glabra roxb. alleviates cisplatin-induced acute kidney injury in mice by activating the Nrf2/HO-1 Signalling Pathway. Phytomedicine.

[76] Jiang J., Shen W., He Y., Liu J., Ouyang J., Zhang C., Hu K. (2024). Overexpression of NLRP12 enhances antiviral immunity and alleviates herpes simplex keratitis via pyroptosis/IL-18/IFN-γ signaling. Int. Immunopharmacol..

[77] He Y., Wang C., Liang Q., Guo R., Jiang J., Shen W., Hu K. (2022). PKHB1 peptide induces antiviral effects through induction of immunogenic cell death in herpes simplex keratitis. Front. Pharmacol..

