# Supplementary material for: Therapeutic Effects of Glycyrrhizic Acid on Dry Eye Disease: Targeting Pyroptosis, Oxidative Stress, and Epithelial Barrier Dysfunction
Source: Int J Mol Sci. 2026 May 6;27(9):4153. doi: 10.3390/ijms27094153 (PMC13163720; doi:10.3390/ijms27094153)
Supplement: Supplementary file 1 [file ijms-27-04153-s001.zip › ijms-4263931-supplementary.pdf]

Supplementary Material

The safety evaluation of GA and SH eye drops in healthy mice had been conducted as part of our original experimental work. In these experiments, healthy mice were treated with GA or SH eye drops (5 μL per eye, twice daily) for 14 consecutive days, followed by ocular surface observation under a stereomicroscope, corneal fluorescein sodium staining, and tear secretion assessment using the phenol red thread test.

The results showed no observable ocular surface abnormalities or damage under stereomicroscopic examination. In addition, no significant changes were detected in corneal fluorescein staining or tear secretion after treatment. These findings suggest that both GA and SH eye drops show favorable ocular surface safety in healthy mice.

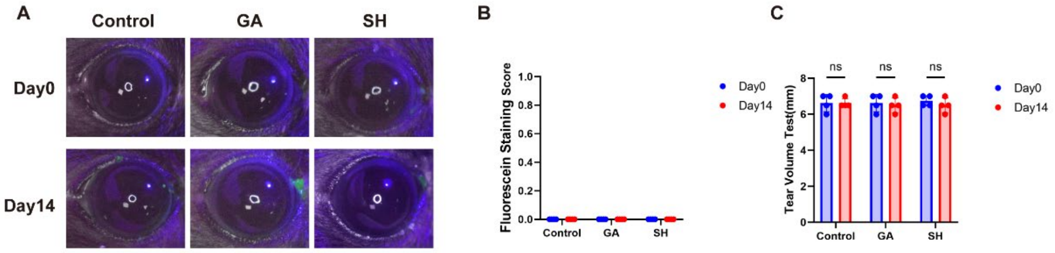

Supplementary Figure S1. (A) Representative photographs of corneal fluorescein sodium staining (CFS) in control, GA and SH groups for 14 days. (B) Quantitative analysis of CFS scores (n = 4). (C) Measurement of tear secretion using the phenol red thread test (n = 4). The data were presented as mean ± SD of at least three independent experiments (ns, p>0.05).

Figure 3

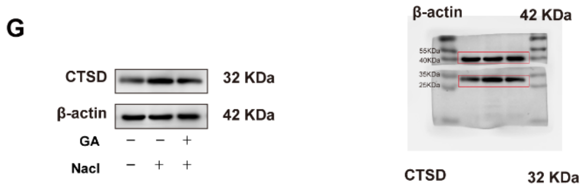

Figure 4

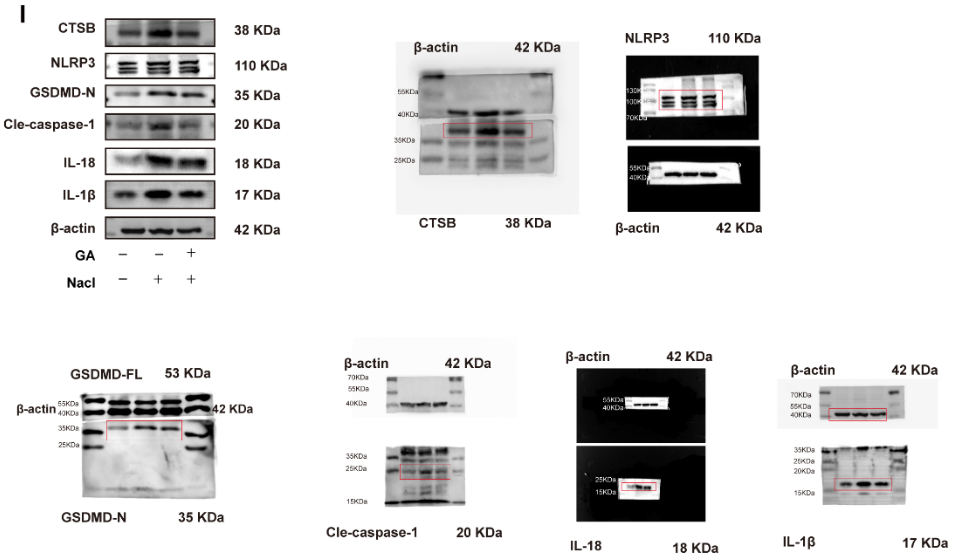

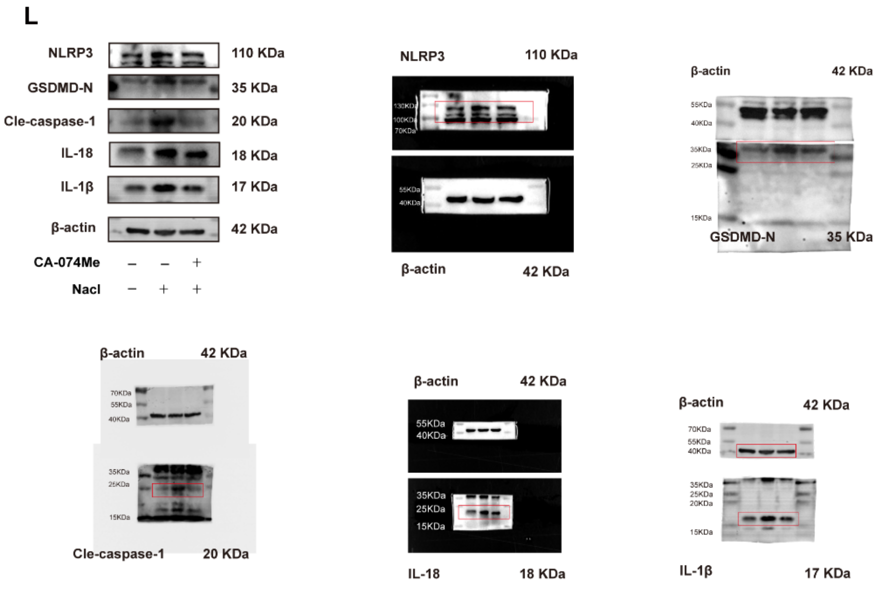

Supplementary Figure S2. The original, uncropped Western blot images of Figure 3G, Figure 4I and Figure 4L.

Figure 4

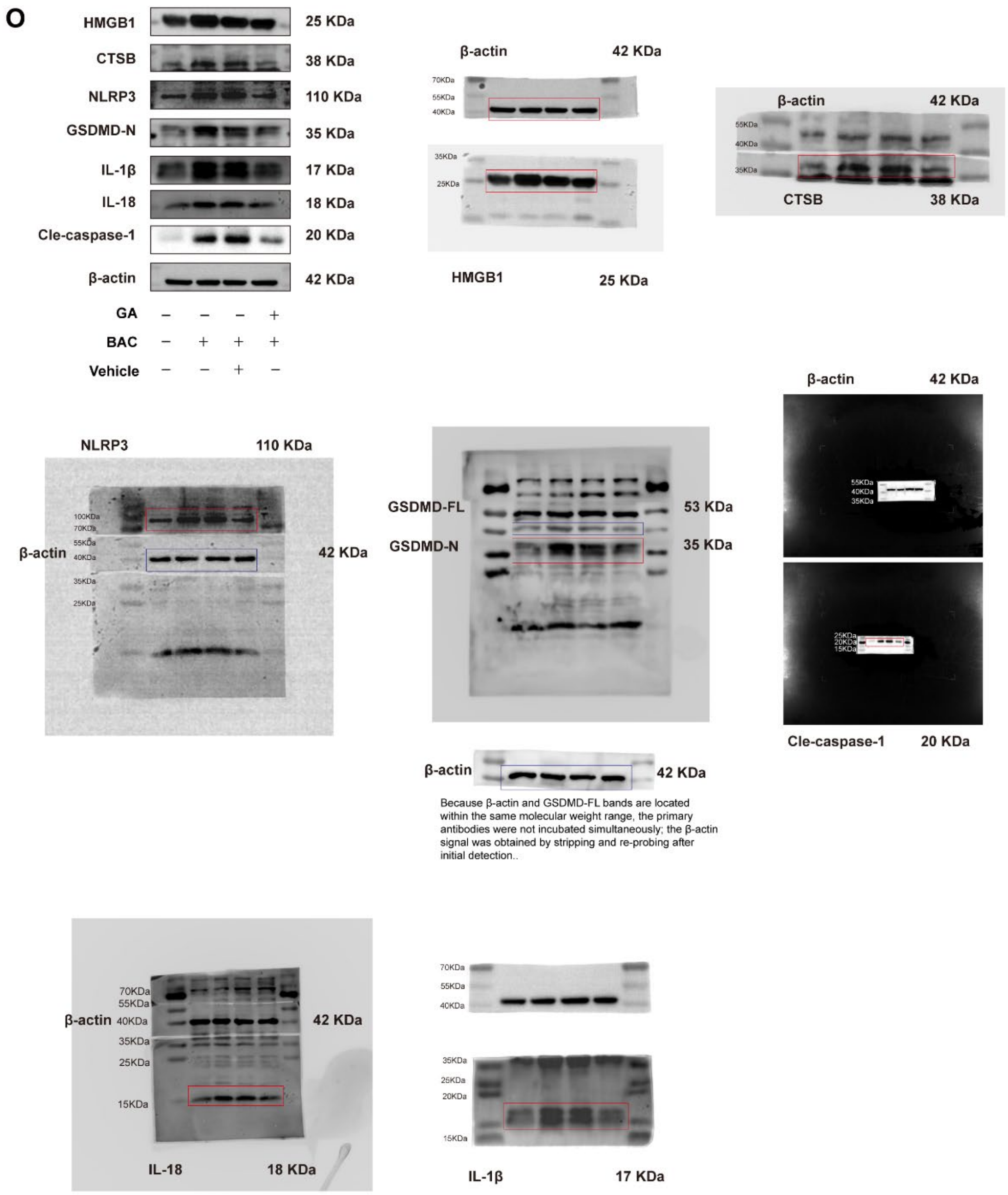

Supplementary Figure S3. The original, uncropped Western blot images of Figure 4O.

Figure 5

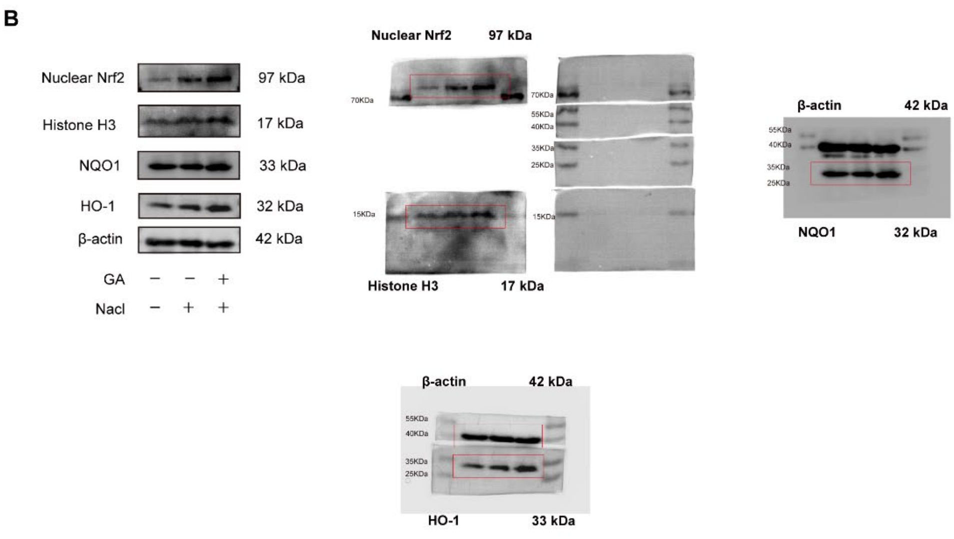

Figure 5

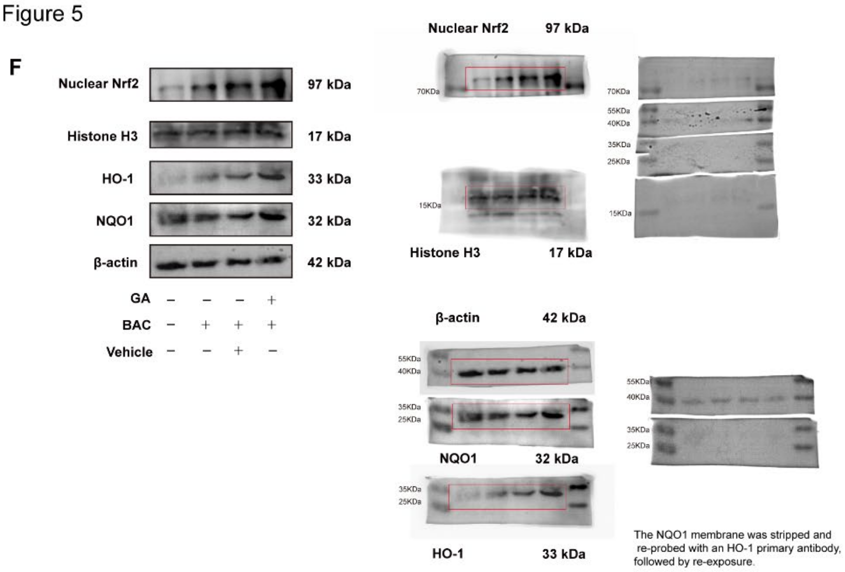

Figure 6

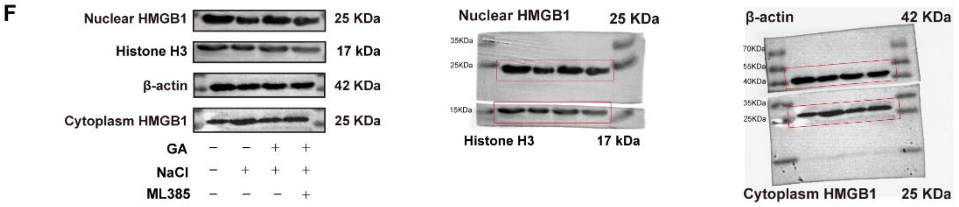

Supplementary Figure S4. The original, uncropped Western blot images of Figure 5B, Figure 5F and Figure 6F.

Figure 7

E

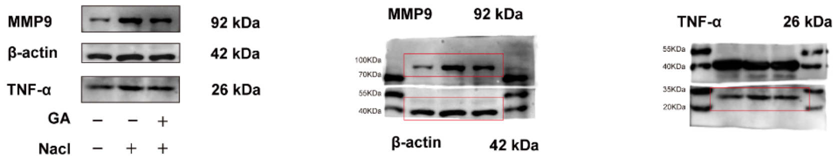

Figure 7

H

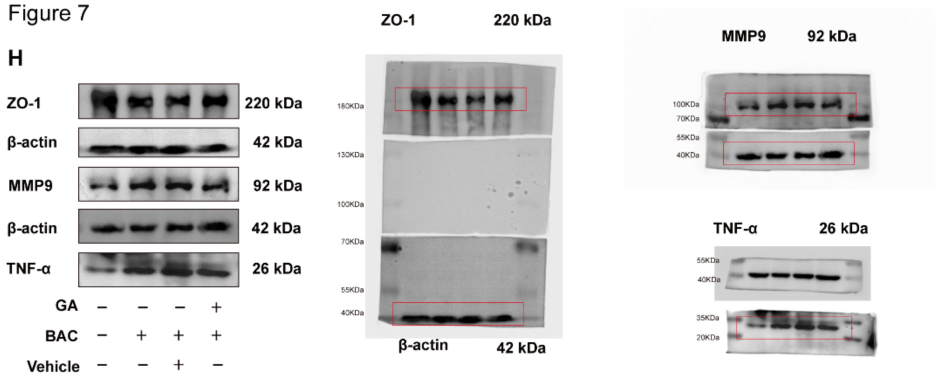

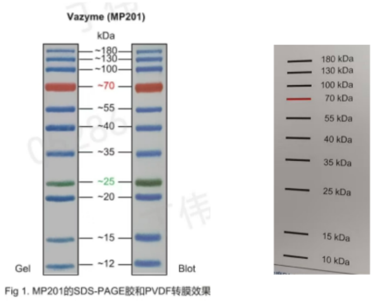

We used two types of protein molecular weight markers for Western blotting: one including a 20 kDa band and one without a 20 kDa band.

Supplementary Figure S5. The original, uncropped Western blot images of Figure 6E and Figure 6H.

NOTE: Please save this file locally before filling in the table, DO NOT work on the file within your internet browser as changes will not be saved. Adobe Acrobat Reader (available free here) is recommended for completion.

The ARRIVE guidelines 2.0: author checklist

The ARRIVE Essential 10

These items are the basic minimum to include in a manuscript. Without this information, readers and reviewers cannot assess the reliability of the findings.

Item		Recommendation	Section/line number, or reason for not reporting
**Study design**	1	For each experiment, provide brief details of study design including:	Lines 115-122; lines 416-432
		a. The groups being compared, including control groups. If no control group has been used, the rationale should be stated.	
		b. The experimental unit (e.g. a single animal, litter, or cage of animals).	Each mouse was considered one experimental unit, and both eyes were included and subjected to the same treatment conditions. For Western blot
**Sample size**	2	a. Specify the exact number of experimental units allocated to each group, and the total number in each experiment. Also indicate the total number of animals used.	Each mouse was considered one experimental unit, and both eyes were included and subjected to the same treatment conditions. For Western blot [illegible]
		b. Explain how the sample size was decided. Provide details of any *a priori* sample size calculation, if done.	**The sample size is consistent with sample sizes reported in similar experimental studies in the field of dry eye disease.**
**Inclusion and exclusion criteria**	3	a. Describe any criteria used for including and excluding animals (or experimental units) during the experiment, and data points during the analysis. Specify if these criteria were established *a priori*. If no criteria were set, state this explicitly.	All data were included in the analysis to avoid selection bias.
		b. For each experimental group, report any animals, experimental units or data points not included in the analysis and explain why. If there were no exclusions, state so.	All data were included in the analysis to avoid selection bias.
		c. For each analysis, report the exact value of *n* in each experimental group.	All data were included in the analysis to avoid selection bias.
**Randomisation**	4	a. State whether randomisation was used to allocate experimental units to control and treatment groups. If done, provide the method used to generate the randomisation sequence.	Lines 422-427
		b. Describe the strategy used to minimise potential confounders such as the order of treatments and measurements, or animal/cage location. If confounders were not controlled, state this explicitly.	Lines 422-427
**Blinding**	5	Describe who was aware of the group allocation at the different stages of the experiment (during the allocation, the conduct of the experiment, the outcome assessment, and the data analysis).	Animals were randomly assigned to experimental groups prior to the study. The investigator responsible for drug administration was aware of group allocation during the conduct of the experiment. However, outcome assessment and data analysis were performed in a blinded fashion
**Outcome measures**	6	a. Clearly define all outcome measures assessed (e.g. cell death, molecular markers, or behavioural changes).	The outcome measures included corneal epithelial integrity, tear secretion, goblet cell density, inflammatory cytokine expression, oxidative stress levels, mitochondrial [illegible]
		b. For hypothesis-testing studies, specify the primary outcome measure, i.e. the outcome measure that was used to determine the sample size.	Given the multifactorial nature of dry eye disease, no single primary outcome was pre-specified. Instead, a set of complementary outcome measures, including corneal fluorescein staining and
**Statistical methods**	7	a. Provide details of the statistical methods used for each analysis, including software used.	Lines 533-538
		b. Describe any methods used to assess whether the data met the assumptions of the statistical approach, and what was done if the assumptions were not met.	Lines 533-538
**Experimental animals**	8	a. Provide species-appropriate details of the animals used, including species, strain and substrain, sex, age or developmental stage, and, if relevant, weight.	Lines 416-432
		b. Provide further relevant information on the provenance of animals, health/immune status, genetic modification status, genotype, and any previous procedures.	Lines 416-432
**Experimental procedures**	9	For each experimental group, including controls, describe the procedures in enough detail to allow others to replicate them, including:	
		a. What was done, how it was done and what was used.	Lines 116-122； lines416-432
		b. When and how often.	Lines 116-122； lines416-432
		c. Where (including detail of any acclimatisation periods).	Lines 116-122； lines416-432
		d. Why (provide rationale for procedures).	Lines 116-122； lines416-432
**Results**	10	For each experiment conducted, including independent replications, report:	
		a. Summary/descriptive statistics for each experimental group, with a measure of variability where applicable (e.g. mean and SD, or median and range).	Lines 533-538
		b. If applicable, the effect size with a confidence interval.	**Effect sizes with confidence intervals were not calculated, as the study was primarily exploratory and based on standard group comparisons.**

The Recommended Set

These items complement the Essential 10 and add important context to the study. Reporting the items in both sets represents best practice.

Item		Recommendation	Section/line number, or reason for not reporting
**Abstract**	11	Provide an accurate summary of the research objectives, animal species, strain and sex, key methods, principal findings, and study conclusions.	Lines 13-32
**Background**	12	a. Include sufficient scientific background to understand the rationale and context for the study, and explain the experimental approach.	Introduction; methods
		b. Explain how the animal species and model used address the scientific objectives and, where appropriate, the relevance to human biology.	Lines 115-122
**Objectives**	13	Clearly describe the research question, research objectives and, where appropriate, specific hypotheses being tested.	Lines 56-58; lines 104-105
**Ethical statement**	14	Provide the name of the ethical review committee or equivalent that has approved the use of animals in this study, and any relevant licence or protocol numbers (if applicable). If ethical approval was not sought or granted, provide a justification.	Lines 574-577
**Housing and husbandry**	15	Provide details of housing and husbandry conditions, including any environmental enrichment.	Six- to eight-week-old female C57BL/6J mice (
**Animal care and monitoring**	16	a. Describe any interventions or steps taken in the experimental protocols to reduce pain, suffering and distress.	All experimental procedures were designed to minimize animal pain, suffering, and distress. Animals were handled gently during all procedures, and topical administration of eye drops was performed carefully to avoid
		b. Report any expected or unexpected adverse events.	No unexpected adverse events were observed during the experimental period. Mild transient ocular irritation immediately after topical administration was occasionally noted, but it resolved spontaneously within a
		c. Describe the humane endpoints established for the study, the signs that were monitored and the frequency of monitoring. If the study did not have humane endpoints, state this.	Humane endpoints were not formally applied in this study; however, animals were closely monitored daily for signs of distress or illness.
**Interpretation/ scientific implications**	17	a. Interpret the results, taking into account the study objectives and hypotheses, current theory and other relevant studies in the literature.	Lines 337-341
		b. Comment on the study limitations including potential sources of bias, limitations of the animal model, and imprecision associated with the results.	Lines 394-411
**Generalisability/ translation**	18	Comment on whether, and how, the findings of this study are likely to generalise to other species or experimental conditions, including any relevance to human biology (where appropriate).	The findings of this study, obtained in a murine model of dry eye disease, may provide insights into the potential therapeutic effects of GA eye drops on ocular surface inflammation and damage. Given the conserved pathological features of dry eye disease across species, including tear film
**Protocol registration**	19	Provide a statement indicating whether a protocol (including the research question, key design features, and analysis plan) was prepared before the study, and if and where this protocol was registered.	A formal study protocol was not registered prior to the study. However, the experimental design, key procedures, and analysis plan were established based on standard practices and previous studies in the field.
**Data access**	20	Provide a statement describing if and where study data are available.	Lines 561-562
**Declaration of interests**	21	a. Declare any potential conflicts of interest, including financial and non-financial. If none exist, this should be stated.	Lines 583-585
		b. List all funding sources (including grant identifier) and the role of the funder(s) in the design, analysis and reporting of the study.	Lines 569-573

Supplementary Tables S1 and S2. the completed ARRIVE checklist

Due to space limitations in the table, brief entries are provided in the checklist, and any content that cannot be fully included is detailed below.

1b Each mouse was considered one experimental unit, and both eyes were included and

subjected to the same treatment conditions. For Western blot analysis, corneas from five Each mouse was considered one experimental unit, and both eyes were included and subjected to the same treatment conditions. mice per group were pooled to obtain sufficient protein, and each pooled sample was treated as one biological replicate.

2a Each mouse was considered one experimental unit, and both eyes were included and subjected to the same treatment conditions. For Western blot analysis, corneas from five Each mouse was considered one experimental unit, and both eyes were included and subjected to the same treatment conditions. mice per group were pooled to obtain sufficient protein, and each pooled sample was treated as one biological replicate.

2b The sample size is consistent with sample sizes reported in similar experimental studies in the field of dry eye disease.

5 Animals were randomly assigned to experimental groups prior to the study. The investigator responsible for drug administration was aware of group allocation during the conduct of the experiment. However, outcome assessment and data analysis were performed in a blinded fashion to minimize bias.

6a The outcome measures included corneal epithelial integrity, tear secretion, goblet cell density, inflammatory cytokine expression, oxidative stress levels, mitochondrial function, and cell death-related markers. At the cellular level, outcomes included cell viability, apoptosis rate, inflammatory gene/protein expression, oxidative stress indicators, and signaling pathway activation.

6b Given the multifactorial nature of dry eye disease, no single primary outcome was pre-specified. Instead, a set of complementary outcome measures, including corneal fluorescein staining and tear secretion, was used to comprehensively assess therapeutic effects.

15 Six- to eight-week-old female C57BL/6J mice (body weight: 22.5 ± 2.5 g) with normal ocular development were used in this study and obtained from the Experimental Animal Center of Yangzhou University. All animals were housed in the Animal Experimental Center of Nanjing Drum Tower Hospital, Affiliated Hospital of Medical School, Nanjing University under specific pathogen-free (SPF) conditions.The housing environment was maintained at a constant temperature of 25°C with a relative humidity of 40–50% under a 12 h/12 h light/dark cycle. Mice were group-housed in standard cages with adequate space, and had free access to standard laboratory chow and water ad libitum throughout the experiment.Prior to experimental procedures, animals were acclimatized to the facility for two weeks. Environmental enrichment was provided through group housing and standard cage conditions to allow normal social and exploratory behaviors. All animals were randomly assigned to experimental groups prior to intervention.

16a All experimental procedures were designed to minimize animal pain, suffering, and distress. Animals were handled gently during all procedures, and topical administration of eye drops was performed carefully to avoid ocular injury. No surgical procedures were involved in this study. Environmental conditions were maintained under standard laboratory housing with free access to food and water to reduce stress.

16b. No unexpected adverse events were observed during the experimental period. Mild transient ocular irritation immediately after topical administration was occasionally noted, but it resolved spontaneously within a short period and did not require intervention. No systemic adverse effects or treatment-related mortality were observed.

18 The findings of this study, obtained in a murine model of dry eye disease, may provide insights into the potential therapeutic effects of GA eye drops on ocular surface inflammation and damage. Given the conserved pathological features of dry eye disease across species, including tear film instability, epithelial injury, and inflammatory responses, the results may have relevance to human ocular surface disease. However, differences between murine models and human disease, as well as the controlled experimental conditions used in this study, should be considered when extrapolating these findings. Further clinical studies are required to confirm the translational applicability of these results.

19 A formal study protocol was not registered prior to the study. However, the experimental design, key procedures, and analysis plan were established based on standard practices and previous studies in the field.
